# ERα-dependent crosstalk between macrophages and cancer cells potentiates vasculogenic mimicry and M2 macrophage polarization in bladder cancer

**DOI:** 10.1186/s12964-025-02297-7

**Published:** 2025-07-15

**Authors:** Qing Liu, Jinpeng Wang, Shan Gao, Zhuolun Li, Wei Zhang, Weiyang Liu, Bingmei Liu, Guanglu Dong, Bosen You

**Affiliations:** 1 Department of Radiation Oncology, The 2nd Affiliated Hospital of Harbin Medical University, Harbin, 150001 China; 2 Department of Urology, The 2nd Affiliated Hospital of Harbin Medical University, Harbin, 150001 China; 3 Department of Pathology, The 2nd Affiliated Hospital of Harbin Medical University, Harbin, 150001 China; 4 The 4th Affiliated Hospital of Harbin Medical University, Harbin, 150001 China

**Keywords:** Bladder cancer, ERα, Vascular mimicry, Polarization of M2 macrophage, Exosome

## Abstract

Gender related differences in the occurrence and progression of bladder cancer are affected by sex hormones and their receptor signaling pathways. Angiogenesis inhibitors targeting vascular endothelial growth factor do not show therapeutic effectiveness in bladder cancer patients. In this study, we found that the expression of ERα is positively correlated with vascular mimicry (VM) formation as well as the infiltration of M2 type tumor associated macrophages (TAM). However, it is unclear how this connection between ERα and macrophages affects VM and its detailed mechanism. Our in vitro results showed that macrophage-induced upregulation of ERα promotes VM in BLCA cells by transcriptionally upregulating CDH5. Further research has found that upregulated ERα in BLCA cells induces polarization of M2 macrophages by activating the PTEN/PI3K/pAKT pathway through exosomes derived from tumor cells. Mechanistic investigations have revealed that exosomal miR-642a-5p upregulated by ERα in BLCA cells downregulate PTEN expression in macrophages by directly targeting the 3’UTR of PTEN mRNA. Preclinical experiments involving in vitro BLCA cell lines and an in vivo mouse xenograft model confirm this newly identified pathway and its feedback circuit, offering new perspectives for the development of innovative treatment strategies for BLCA.

**Supplementary Information:**

The online version contains supplementary material available at 10.1186/s12964-025-02297-7.

## Introduction

Bladder cancer (BLCA) ranks as the second most common malignancy in the urinary system, second only to prostate cancer (PCa) [1]. Although the incidence of BLCA is approximately three times higher in men than in women, the prognosis for female BLCA patients at advanced stages is notably worse than that for male patients [2, 3]. This gender-based difference has prompted us to reexamine and explore the potential role of the female hormone estrogen and its associated estrogen receptors (ERs) in the development of BLCA. According to previous studies, ERβ acts as an oncogenic factor in BLCA, driving disease progression, while ERα has been considered as a tumor-suppressive factor that prevents tumor initiation [4]. Nevertheless, despite ERα’s protective role in BLCA initiation, evidence suggests that higher ERα expression is associated with tumor aggressiveness and unfavorable patient outcomes [5]. Therefore, it is important to reevaluate and reassess the underlying roles of ERα in BLCA, considering the dynamic changes in biological behavior and the intricate tumor microenvironment associated with tumor progression.

Macrophages infiltrating the tumor microenvironment (TME) of solid tumors are named tumor-associated macrophages (TAMs). TAMs play a pivotal role in the inflammatory response and the tumor microenvironment [6]. TAMs exhibit characteristics that support tumor growth, such as promoting the development of blood vessels (angiogenesis), suppressing the immune response, creating a niche for cancer stem cells, influencing the remodeling of the extracellular matrix, and facilitating epithelial-mesenchymal transition (EMT) [7–9]. Evidence from epidemiological and clinical studies confirms that TAM infiltration is strongly associated with poor prognosis in BLCA [10–12]. In BLCA, elevated levels of TAMs within tumors may correlate with a higher risk of recurrence and can hinder the effectiveness of BCG treatment, a common therapy for this cancer [13]. Interestingly, prior research has unveiled that targeting estrogen/ERα can enhance the effectiveness of BCG treatment in BLCA [14]. Studies conducted on other solid tumors have also shed light on the significance of ERα-mediated interactions between tumor cells and macrophages, which can promote tumor development and suggest potential new therapeutic approaches [15–18]. Nonetheless, it remains uncertain whether ERα’s involvement in the interaction between macrophages and BLCA cells contributes to tumor progression.

In this study, we discovered a new role of ERα in the way TAMs and BLCA cells interact, which leads to the promotion of VM formation and M2 macrophage polarization, and also examined the underlying molecular mechanisms. Our research presents a new perspective, suggesting that ERα contributes to pro-tumor effects by shaping the BLCA tumor environment, which may help explain why female BLCA patients often have a poor prognosis and could open up new avenues for potential treatments.

## Materials and methods

### Cell culture

BLCA T24 and TCC-SUP cell lines, monocytic leukemia cell line THP-1 and the HEK 293T cell line were purchased from the American Type Culture Collection (ATCC, Manassas, VA). T24, TCCSUP, HEK 293T cells and THP-1 cells were cultured in DMEM and RPMI-1640 Medium, supplemented with 10% fetal bovine serum (FBS), antibiotics (100 units/ml penicillin, 100 mg/ml streptomycin), and 2 mM glutamine (Invitrogen, Grand Island, NY, USA). All cell lines were maintained in a humidified 5% CO_2_ environment at 37 °C.

### Co-culture experiment

THP-1 cells were treated with 100ng/ml PMA (Sigma) for 48 h to differentiate into macrophages. THP-1-induced macrophages were seeded in the transwell chambers with 0.4 μm pore size (Corning Life Science) and BLCA cells in the bottom of a 6-well plate. The co-culture was maintained for 2 days, after which both BLCA cells and the conditioned medium (CM) were harvested for further experimentation.

### Lentivirus packaging and transfection

The lentiviral vector pLKO.1 was used to construct shRNA plasmids for knockdown, while pWPI was used for overexpression. Both plasmids were co-transfected with the packaging plasmid psPAX2 and the envelope plasmid pMD2.G into HEK293T cells for 48 h using the standard CaCl_2_ transfection method to generate lentivirus particles, which were subsequently dissolved in medium, filtered and either utilized immediately or stored at -80 °C for future use.

### Protein extraction and Western blot

The cells were lysed using RIPA buffer supplemented with PMSF (1:1000) and cocktail (1:100). Protein samples (30–50 µg) were equally loaded, boiled and separated on 6–10% SDS/PAGE gels, then transferred onto PVDF membranes (Millipore, Billerica, MA). Blocking was performed using 5% skimmed milk dissolved in TBST solution, followed by a 1-hour incubation with specific primary antibodies and then HRP-conjugated secondary antibodies. The ECL system (Thermo Fisher Scientific, Rochester, NY) was used to visualize and assess the blots.

### RNA extraction and quantitative real-time PCR (qRT-PCR) analysis

Total RNAs were extracted using Trizol reagent (Invitrogen, Grand Island, NY), followed by reverse transcription of 2 µg total RNA using Superscript III transcriptase (Invitrogen, Grand Island, NY). Quantitative real-time PCR (qRT-PCR) was conducted on a Bio-Rad CFX96 system with SYBR green to determine the mRNA level of a gene of interest. Expression levels were normalized to GAPDH mRNA using the 2^−ΔΔ^Ct method. The primers used are listed in Table *S1.*

### 2D Matrigel-based tube formation assay

Growth factor-reduced Matrigel (BD Biosciences, USA) was allowed to melt at 4°C overnight and was then added to a 96-well plate at a volume of 50ul per well. The plate was incubated at 37 °C for one and a half hours, and the cells were resuspended in serum-free DMEM at a final concentration of 2.5 × 10^4^ cells/100ul and added to the wells coated with Matrigel. After 4–6 h of incubation at 37 °C, tube formation was observed using a microscope (Olympus, Tokyo, Japan). The length of tubules in each field of view was photographed, and 3–5 random fields in each well were counted to obtain an average length. Tubules were quantified using ImageJ software, as described in a previous study [19].

### 3D collagen 1-induced tube formation assay

Cells suspended in DMEM medium with final concentration were added into soluble rat tail type I collagen in acetic acid (Corning, Corning, NY) mixed with 10x reconstitution buffer,1:1(v/v) and 1 M NaOH (pH 7, 10–20 µl) [20]. The 200 µl mixture was then loaded into a 48-well culture plate and placed in a cell incubator at 37ºC for 5–7 days.

### Chromatin Immunoprecipitation (ChIP)

Briefly, cell lysates were pre-cleaned using protein A-agarose conjugated normal IgG (sc-2027, Santa Cruz), then an anti-ERα antibody (2.0 µg) (D8H8, Cell Signaling Technology) was added and incubated overnight at 4 °C. In the negative control, IgG was used. Specific primer sets designed for amplifying the target sequence within the CDH5 gene’s promoter were used for qPCR, and the resulting PCR product was analyzed using agarose gel electrophoresis.

### Luciferase reporter assay

The human CDH5 promoter was cloned into PGL3 basic vectors (Promega), the ERα binding site within the promoter region was mutated, and the mutant promoter was cloned into the PGL3 vector. As a baseline control, the internal control pRL-TK was used. Cells were seeded in 24-well plates and transfected with the respective cDNAs using Lipofectamine 3000 (Invitrogen, Carlsbad, CA) following the manufacturer’s instructions. After 36–48 h of transfection, Luciferase activity was assessed using the Dual-Luciferase Assay (Promega). For the 3’UTR of PTEN, versions with both wild-type and mutant miRNA-responsive elements were inserted into the psiCHECK-2 vector (Promega). Then, the cells were plated in 24-well plates and transfected with the constructed cDNAs, excluding pRL-TK, and the subsequent steps were performed as described above.

### Exosomes isolation and identification

BLCA cells were cultured in a regular DMEM medium with 10% exosomes-depleted FBS for 3 days. Then, the cultured medium was collected and centrifuged at 1300 rpm for 10 min to obtain the supernatant, which was subsequently treated with a 0.45 μm filter, followed by centrifugation at 9600 rpm and 4 °C for 20 min and filtered through a 0.2 μm filter to remove any remaining cells and debris. The resulting supernatant was ultracentrifuged at 25,800 rpm for 70 min using a Beckman Coulter instrument to collect the pellet, which contained exosomes along with some contaminating proteins. To obtain purified exosomes, a 20 ml volume of sterilized PBS was used to wash the pellet, followed by repeating the ultracentrifugation step. The final pellet contained the exosomes, which were resuspended in PBS or cell lysis buffer for subsequent experiments. To confirm the presence of exosomes, electron microscopy was used for visualization. Electron micrographs were captured using a JEOL, JEM-1230 (Tokyo, Japan) transmission electron microscope operating at an excitation voltage of 80 kV. Additionally, western blot analysis was conducted to detect the exosomal markers CD9 and CD63.

### Patients and samples

A total of 40 histologically confirmed BLCA tissue samples were collected between April 1, 2021, and September 1, 2023, from the Department of Pathology at the Second Affiliated Hospital of Harbin Medical University (Harbin, China). Cases that were previously treated with neoadjuvant chemotherapy or radiotherapy were excluded. After the patient signs the scientific ethics consent form, all samples collected for the study are fixed in 10% formalin and then embedded in paraffin. Our research protocol was approved by the Institutional Ethics Review Board of the hospital.

### In vivo studies

Female athymic BALB/c nude mice, aged 4–6 weeks, were obtained from the Medical Laboratory Animal Center of Harbin Medical University (Harbin, Heilongjiang, China). In the first part of the animal study, a mixture of TCC-SUP cells (5 × 105) and THP-1 cells (5 × 105), combined with Matrigel (1:1), was subcutaneously injected into the left hip of each mouse. Subsequently, the tumor-bearing mice were randomly divided into four groups, each comprising 6 mice. Two of these groups received intravenous injections of PBS every 3 days, while the remaining two groups were administered exosomes derived from macrophage co-cultured TCC-SUP cells. After 2 weeks of inoculation, a 4-week treatment regimen was initiated, involving intraperitoneal injections of ICI-182,780 (2.5 mg/kg) or DMSO administered every other day until the mice reached the predetermined treatment endpoints. Following this, the mice were euthanized, and their tumors were removed for measurement and further analysis. In the second part of the animal study, the mice were randomly divided into three groups: TCC-SUP group, TCC-SUP-pLKO + THP-1 group, and TCC-SUP-miR642a-5p inhibitor + THP-1 group, each comprising 6 mice. TCC-SUP cells (5 × 105) were subcutaneously injected either alone or mixed with TCC-SUP-pLKO or TCC-SUP-miR642a-5p inhibitor (5 × 105), along with THP-1 cells (5 × 105), using Matrigel (1:1) into the left hip of each mouse. After six weeks, the mice were euthanized, and the transplanted tumors were removed for measurement and further analysis. Throughout the study, the average gross tumor sizes of each group were monitored weekly using calipers to calculate changes in volume, following the formula: [(short axis^2 × long axis)/2]. All mice were maintained under pathogen-free conditions in the animal facility at Harbin Medical University (Harbin, China). All animal experiments were approved and supervised by the Harbin Medical University Institutional Animal Use and Care Committee.

### Statistical analysis

Experiments were repeated independently at least 3 times, with data points obtained in triplicate. The results are presented as mean ± standard deviation (S.D). Statistical significance was assessed using Student’s t-test and two-way ANOVA, performed with SPSS 22 (IBM Corp., Armonk, NY) or GraphPad Prism 6 (GraphPad Software, Inc., La Jolla, CA). P-values less than 0.05 were considered statistically significant (*, *P* < 0.05, **, *P* < 0.01, ***, *P* < 0.001).

## Results

### The expression of ERα is positively correlated with TAMs infiltration in BLCA

**Fig. 1:**
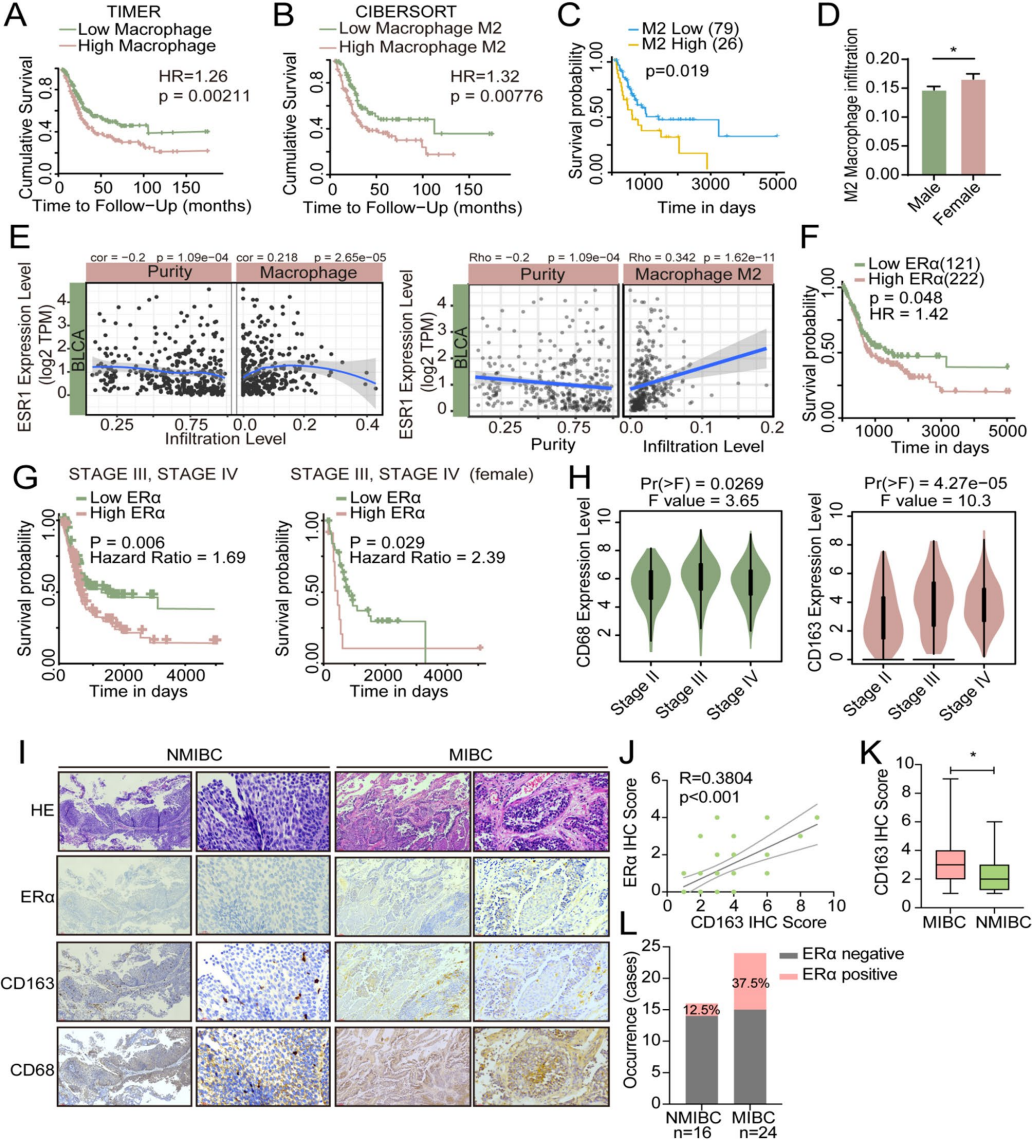
The expression of ERα is positively correlated with macrophage infiltration in BLCA. (**A**) Survival analysis of macrophage infiltration levels in BLCA patients. (**B**) Survival analysis of M2 macrophage infiltration levels in BLCA patients. (**C**) Prognostic value of M2 macrophage infiltration level in female BLCA patients. (**D**) Analysis of M2 macrophage infiltration levels in female and male BLCA patients. (**E**) Correlation between ESR1 gene expression and the level of macrophage infiltration (left) or M2 macrophage infiltration (right) in BLCA patients. (**F**) The prognostic value of ERα expression in BLCA patients analyzed using the TCGA database. (**G**) Prognostic value of ERα expression in BLCA patients (left) or female BLCA patients (right) in stage III and IV disease. (**H**) Expression of CD68 (Left) and CD163 (Right) in TCGA samples at different stages of BLCA. (**I**) IHC comparison of CD163/CD68 and ERα in human BLCA tissue samples. (**J**) The correlation between ERα and CD163 expression in human BLCA tissue samples. (**K**) Expression of CD163 in MIBC and NMIBC human samples. (**L**) Our statistical analysis of the occurrence of cases positive for ERα expression in MIBC and NMIBC human samples. *Data is presented as the mean ± SD*, *p < 0.05, **p < 0.01

Macrophages constitute a prominent group of infiltrating immune cells in various solid tumors, including BLCA. To investigate the role of macrophages within the tumor microenvironment and their impact on BLCA progression, we initially assessed the prognostic significance of macrophage infiltration using TIMER2.0 [21]. Our analysis revealed that BLCA patients exhibiting high levels of macrophage infiltration experienced poorer survival outcomes compared to those with lower levels of macrophage infiltration (Fig. 1A). A similar pattern was observed when evaluating the prognostic value of M2 macrophage infiltration(Fig. 1B). Given the disparities in BLCA incidence and prognosis between genders, we further scrutinized the prognostic relevance of M2 macrophage infiltration in male and female BLCA patients separately. Among female patients, those with elevated levels of M2 macrophage infiltration exhibited worse survival outcomes compared to their counterparts with lower levels (Fig. 1C), while in male patients, no significant difference was observed between the two groups (Sfig.1 A). Furthermore, our analysis unveiled that female BLCA patients displayed higher levels of M2 macrophage infiltration than their male counterparts (Fig. 1D). We proceeded to investigate the correlation between hormone receptors and macrophage infiltration using TIMER2.0. Our findings demonstrated a positive correlation between ESR1, the transcript gene of ERα, and macrophage infiltration as well as M2 macrophage infiltration in BLCA (Fig. 1E). However, M2 macrophage infiltration did not exhibit significant correlations with either ESR2 or AR expression (Sfig.1B).

By applying the online tool TRGAted, we analyzed the association between ERa expression and the survival of BLCA patients. According to TRGAted analysis results, BLCA patients with high ERα expression had poorer survival rates(Fig. 1F) [22]. Among patients with advanced BLCA in stage III and IV, patients with higher ERα expression had worse survival rates, especially female patients (Fig. 1G). However, in patients with BLCA at earlier stages (stage I and II), higher expression levels of ERα were associated with better survival (Sfig. 1 C). The above results suggested that ERα may play a different role in advanced BLCA compared with early-stage BLCA.

The expression of TAMs marker CD68 and CD163 was analyzed according to different clinical stages based on TCGA datasets. The results showed that the expression of these two markers was significantly increased in patients with stage III and IV compared to those in stage II (Fig. 1H). According to the results of our analysis of GSE13507, the expression of CD163 in primary BLCA tumors and recurrent tumors was positively correlated with the expression of ESR1 and was statistically significant (Sfig.1D). To elucidate the correlation between TAMs infiltration and ERα expression in BLCA, we detected the expression of CD68/CD163 and ERα in 40 BLCA human samples, including 24 muscle-invasive bladder cancer (MIBC) samples and 16 non-muscle-invasive bladder cancer (NMIBC) samples, and the results showed that the expression of ERα was positively correlated with TAMs infiltration (Fig. 1I-J). We also found that CD163 expression was significantly higher in MIBC than in NMIBC samples(Fig. 1K). The proportion of ERα-positive samples in MIBC is significantly higher than that in NMIBC(Fig. 1L).

### TAMs increases ERα expression and promotes tumor cell vasculogenic mimicry

Our previous studies showed that TAMs promote VM formation in renal cell carcinoma [23]. VM has been considered to be related to cancer invasion and metastasis and insensitivity or resistance to anti-vascular therapy [24, 25]. To investigate the relationship between TAMs and VM in BLCA, an in vitro co-culture system was established according to the methods in our published paper [23]. First, we examined the expression levels of ERα and the ability to form VM in four different bladder cancer cell lines (J82, TCC-SUP, T24 and UMUC-3). Results showed that the ERα expression level and VM formation ability of the TCC-SUP cell line were stronger than those of the other three cell lines, followed by UMUC-3 cells. The ERα expression level and VM formation ability of T24 cells are weaker than those of UMUC-3 and TCC-SUP cells, and J82 cells do not have the ability to form tubes in in vitro tests (Sfig.2 A-B). Therefore, we selected TCC-SUP cells and T24 cells for subsequent experiments.

**Fig. 2:**
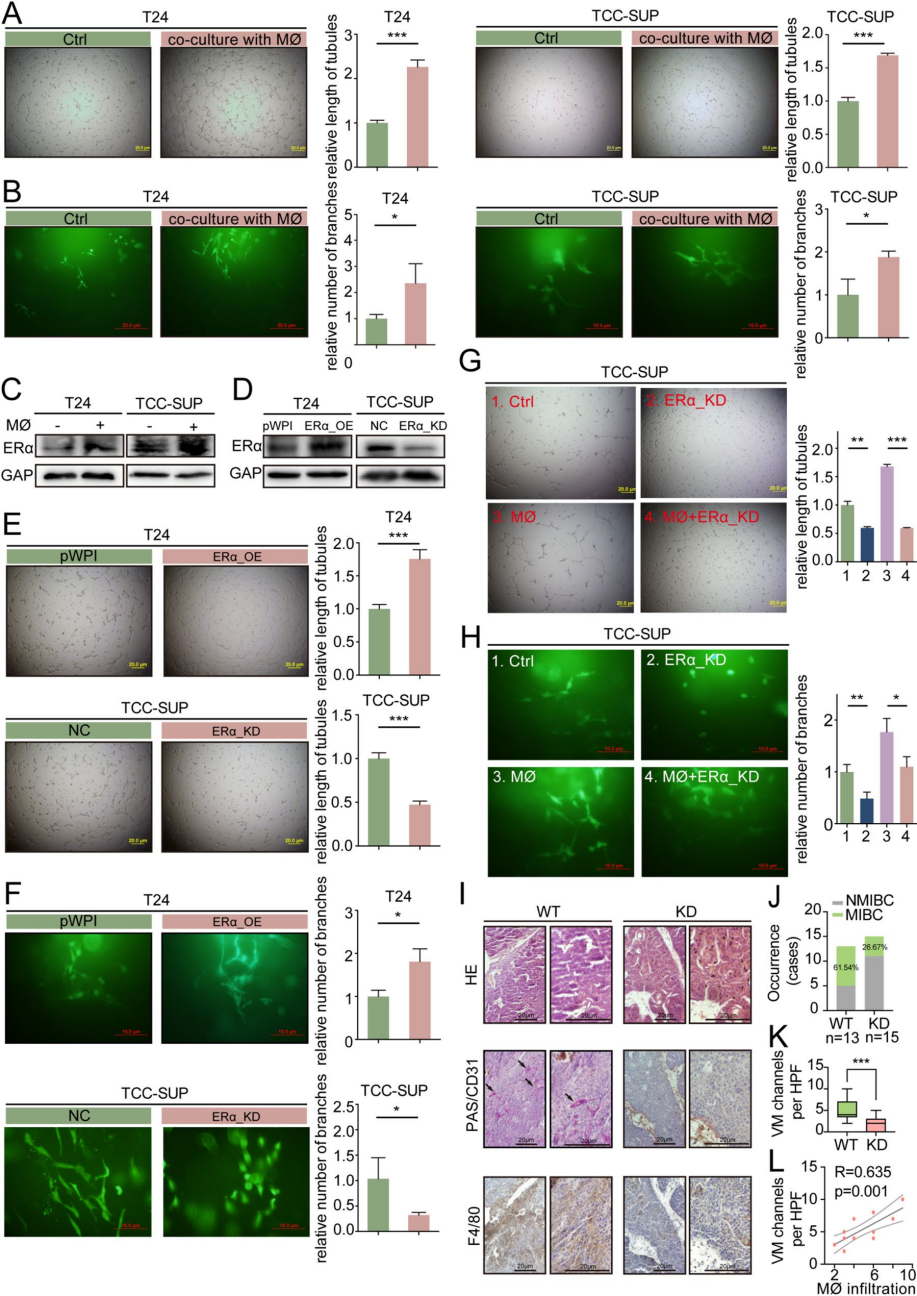
Macrophages increased ERα expression and promoted VM formation in BLCA cells. (**A-B**) Matrigel-coated 2D VM formation assay (**A**) and collagen-based 3D VM formation assay (**B**) were used to test the ability of VM formation in T24 and TCC-SUP cells with/without macrophages co-cultured. (**C**) Western blot assay for detecting ERα protein levels in T24 cells (left) and TCC-SUP cells (right) with/without macrophages co-cultured. (**D**) Western blot was performed to detect ERα expression in ERα-knockdown (ERα-KD) TCC-SUP cells (left) and ERα-overexpressing (ERα-OE) T24 cells (right). (**E-F**) 2D and 3D VM formation assay for testing the ability of VM formation in TCC-SUP cells transfected with NC/ERα-KD virus and in T24 cells transfected with NC/ERα-OE virus. (**G-H**) 2D and 3D VM formation assays were performed in TCC-SUP cells transfected with ERα-KD virus with/without macrophages co-cultured. (**I**) IHC staining of mouse macrophage-specific marker F4/80 and VM marker PAS(+)/CD31(-) in BLCA tissues of ERα-KO and WT mice. Black arrows indicate the VM channels surrounded by tumor cells. (**J**) The incidence of MIBC cases in ERα-KO and WT mice. (**K**) Comparison of the VM channels per high-quality frame (**HPF**) in ERα-KO and WT mice. (**L**) The correlation between VM formation and macrophage infiltration. *For. ****A***,*** B ***and ***E-H***, *quantitation is shown in the graph at right. Data is presented as the mean ± SD*, *p < 0.05, **p < 0.01, ***p < 0.001

As shown in Fig. 2A and B, co-culturing with macrophages significantly enhanced the ability of T24 and TCC-SUP cells to form both 2D and 3D VM structures. We assessed the expression of ERα in the BLCA cells after co-culture with macrophages, which revealed a significant increase in ERα expression (Fig. 2C). To validate the role of ERα in macrophage-induced VM formation, we conducted experiments to assess both 2D and 3D VM formation abilities. We compared TCC-SUP cells transfected with lentivirus generated from ERα-knockdown vector (ERα-KD) with TCC-SUP cells transfected with lentivirus from normal control vector (NC). T24 cells transfected with lentivirus generated from ERα-cDNA-vector (ERα-OE) were compared with T24 cells transfected with lentivirus from pWPI-cDNA-vector (NC). Results demonstrated that reducing the expression of ERα hinders the formation of 2D and 3D VM, while increasing its expression promotes VM formation (Fig. 2D-F).

To investigate whether macrophages enhance VM formation by upregulating ERα expression, rescue experiments were conducted. TCC-SUP cells were transfected with ERα-KD lentivirus, then co-cultured with or without macrophages. Subsequently, tumor cells were harvested for 2D/3D VM formation assays. Results indicated that knockdown of ERα reverses the enhanced VM formation induced by co-culturing with macrophages (Fig. 2G-H). In addition, we performed rescue experiments using the estrogen receptor inhibitor ICI-182,780. The results showed that inhibinting ERα by ICI-182,780 could also reverses the enhanced VM formation induced by co-culturing with macrophages (Sfig. 2 C). Further experiments were performed to examine the presence of the macrophage marker F4/80 and the VM marker PAS+/CD31- in BBN-induced BLCA tumors from ERα knockout (ERα-KO) and wild-type (WT) mice (Fig. 2I). The proportion of MIBC in WT samples was significantly higher than that in ERα-KO group (Fig. 2J). Our results showed that VM formation was significantly reduced in ERα-KO tumors compared with WT tumors (Fig. 2K) and there was a positive correlation between VM formation and macrophage infiltration (Fig. 2L).

Taking together, results from Fig. 2A-L and Sfig.2 A-C demonstrated that ERα acts as a mediator of TAMs-promoted VM formation.

### ERα promotes VM formation by upregulating CDH5

**Fig. 3:**
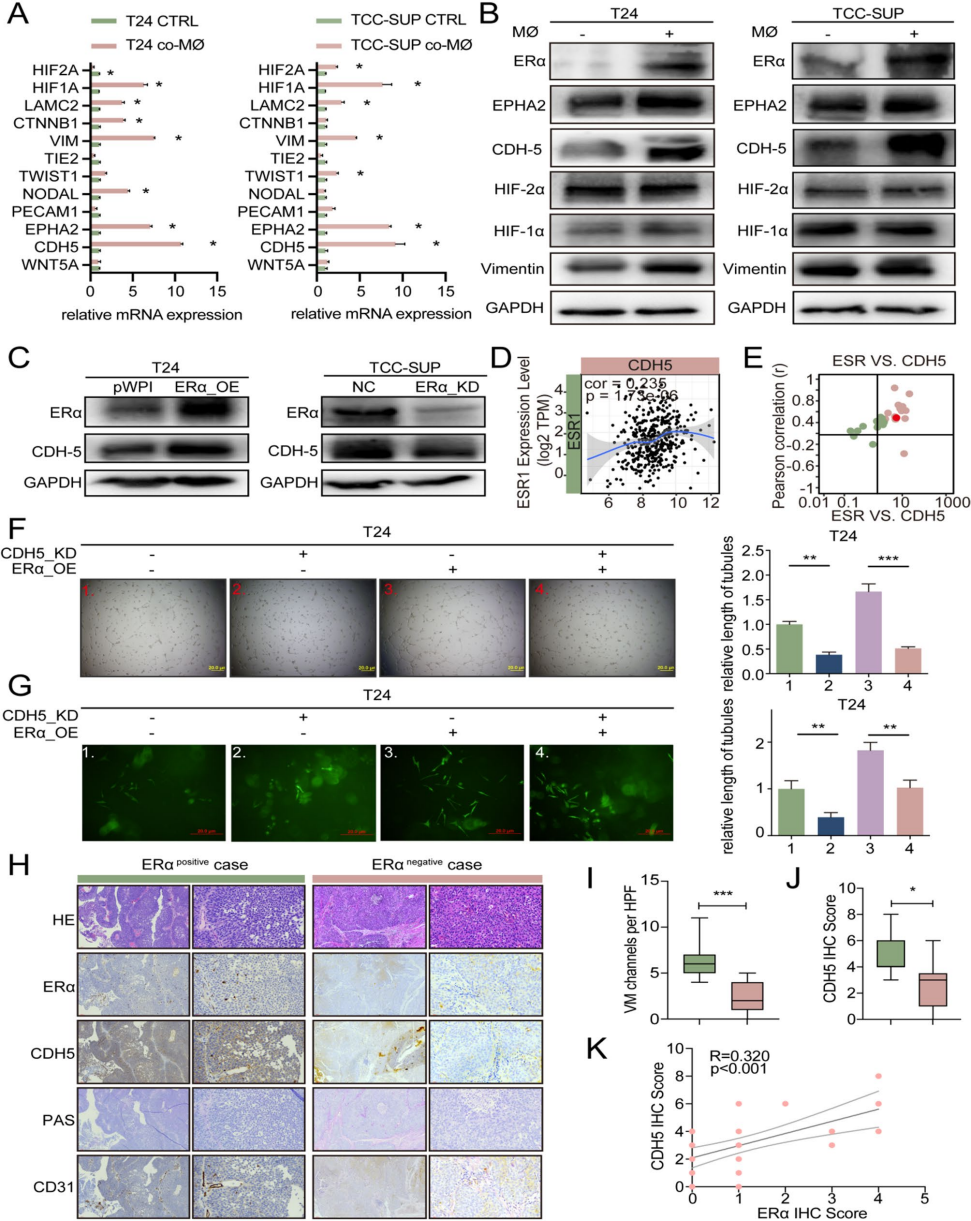
ERα promotes VM formation by upregulating CDH5. (**A**) The qRT-PCR assays for testing the 12 VM-related genes in T24 (left) and TCC-SUP (right) cells co-cultured with macrophages versus the control. (**B**) Western blot was performed to detect the 5 selected genes based on results from Fig. 3A. (**C**) Western blot was performed to detect ERα and CDH5 expression in ERα-overexpressing (ERα-OE) T24 cells (left) and ERα-knockdown (ERα-KD) TCC-SUP cells (right). (**D**) Correlation between CDH5 and ESR1 gene expression in BLCA. (**E**) Correlation between CDH5 and ESR1 gene expression in pan-cancer. (**F-G**) 2D and 3D VM formation assays were performed in T24 cells transfected as indicated in the upper panel. (**H**) IHC comparison of CDH5, ERα and PAS(+)/CD31(-) in ERα-positive expression group (*n* = 11) and ERα negative expression group (*n* = 29). (**I**) Comparison of VM channels per high-quality frame (HPF) in ERα-positive and ERα-negative groups. (**J**) Comparison of expression levels of CDH5 in ERα-positive and ERα-negative groups. (**K**) The correlation between the expression levels of CDH5 and ERα. *For****F****and****G***, quantitation is shown in the graph at right.Data are presented as means ± SD. *p < 0.05, **p < 0.01,* ns = no significance compared with the control*

To elucidate the mechanisms underlying how macrophage-induced ERα promotes VM formation, we identified twelve well-known VM-related molecules at both the mRNA level and protein level in T24 and TCC-SUP cells, whether cocultured with macrophages or not (Fig. 3A-B). The results revealed a significant increase in CDH5 expression in co-cultured T24 and TCC-SUP cells. To further investigate whether ERα can regulate CDH5, additional experiments were conducted. These experiments demonstrated that knockdown of ERα expression led to a decrease in CDH5 expression while overexpression of ERα had the opposite effects (Fig. 3C). Based on the online TCGA database [26], we analyzed the correlation between ESR1 and CDH5 in BLCA and found that there is a significant positive correlation between these two genes (Fig. 3D). Additionally, we examined the correlation between ESR1 and CDH5 across 34 different cancer types, which showed a significant positive correlation in most cancer types, including BLCA (Fig. 3E).

To confirm whether ERα promotes VM formation by regulating CDH5, rescue experiments were conducted, and the results demonstrated that knockdown of CDH5 expression in T24 cells reduced VM formation and reversed overexpression of ERα-induced VM formation (Fig. 3F, G). Conversely, overexpression of CDH5 in TCC-SUP cells enhanced VM formation and reversed knockdown of ERα-suppressed VM formation (Sfig. 3 A-B). Next, we used the online tool UALCAN to analyze the survival of BLCA patients based on the TCGA database, which showed that patients with high CDH5 expression had a poor prognosis, especially female patients (Sfig. 3 C). Furthermore, CDH5 expression was significantly higher in tumor tissues from patients at late (III and IV) stages compared to those at early (II) stages (Sfig. 3D). Next, we detected the ERα expression via IHC in BLCA samples, then separated patients into the ERα-positive group and the ERα-negative group (Fig. 3H). As shown in Fig. 3I-J, the expression of CDH5 and the formation of VM were much higher in ERα-positive tumor tissues than in ERα-negative tumor tissues. Moreover, the expression level of ERα was positively correlated with the level of CDH5 (Fig. 3K).

Taken together, results presented in Fig. 3A-K and Sfig. 3A-D suggest that ERα function to affect VM formation by modulating CDH5 expression.

### Mechanism dissection of how ERα regulates CDH5 expression *via* transcriptional regulation

**Fig. 4:**
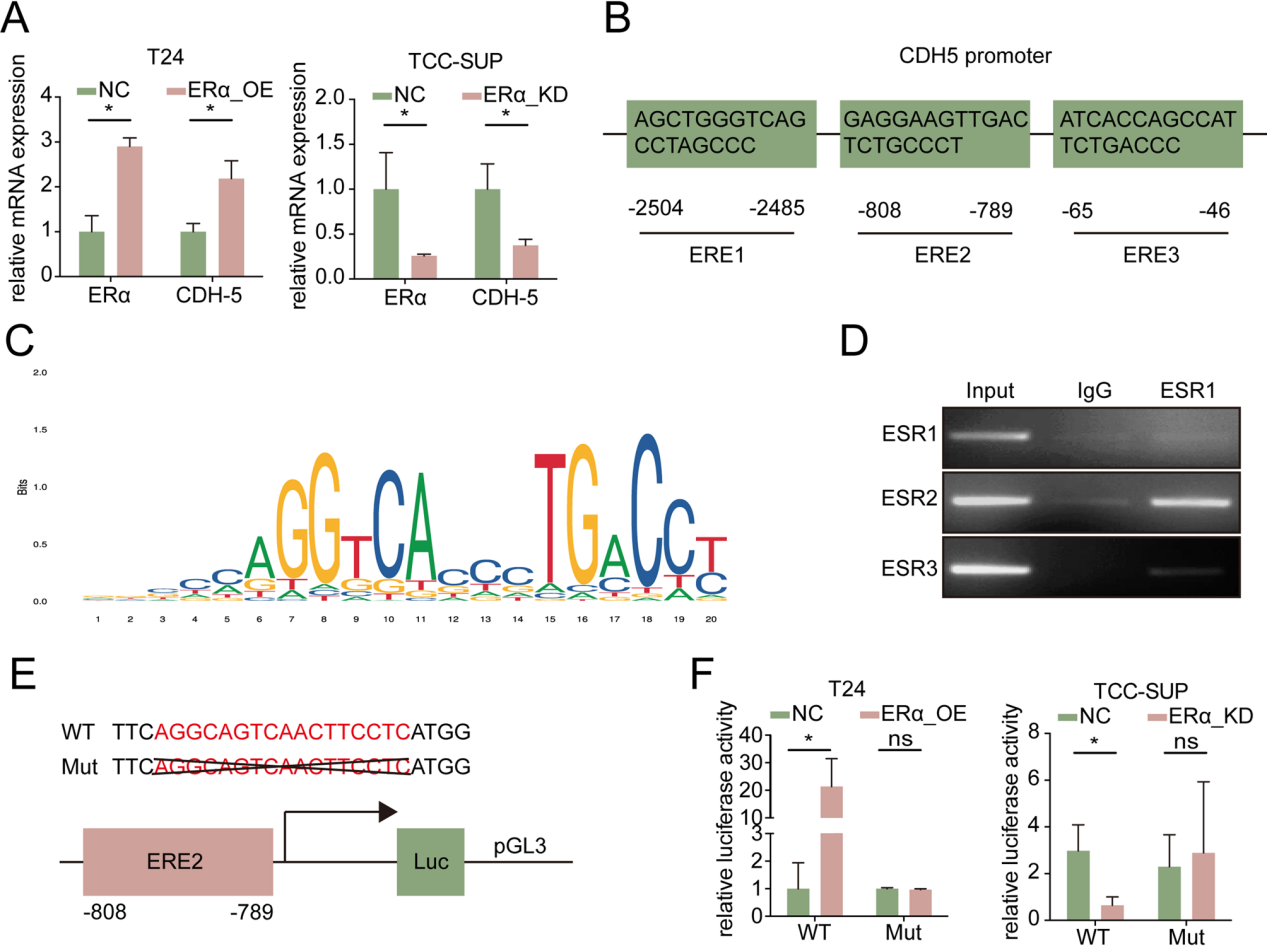
ERα transcriptionally upregulated CDH5 expression. (**A**) qRT-PCR assays for detecting ERα and CDH5 in T24 cells overexpressed with ERα (left) and TCC-SUP cells transfected with ERα-knockdown virus (right). (**B**) Illustration of the predicted EREs on CDH5 promoter region. (**C**) ERE motif sequence. (**D**) CHIP assay was performed to detect the binding of ERα to ERE2 in the CDH5 promoter region. (**E**) The pGL3 reporter plasmids containing the wild-type (WT) or mutant (Mut) ERE2 were designed to perform luciferase reporter assay. (**F**) Luciferase reporter activity was measured and analyzed after transfection of WT or Mut ERE2 of the CDH5 promoter into T24 cells with NC/ERα-OE (left) and TCC-SUP cells with NC/ERα-KD (right). Data are presented as means ± SD. *p < 0.05, **p < 0.01,* ns = no significance compared with the control*

To investigate how ERα regulates CDH5 in BLCA cells, we examined the mRNA levels of CDH5 in TCC-SUP-ERα-KD versus TCC-SUP-NC cells and T24-ERα-OE versus T24-NC cells. The results showed that changes in CDH5 mRNA levels were consistent with changes in its protein expression (Fig. 4A), indicating that transcriptional regulation could be the primary mechanism. Next, ChIP assay was conducted to determine whether ERα can bind to the CDH5 promoter and regulate its transcription. Three predicted ESR1 response elements (EREs) were identified in a 3-kb region on the CDH5 promoter, which could potentially function through ERα binding (Fig. 4B, C). The ChIP results demonstrated that ERα binds to ERE2 in the CDH5 promoter region to initiate gene transcription (Fig. 4D). Then, we performed luciferase assays using pGL3 reporter plasmids containing either the wild-type (WT) or mutant (Mut) ERE2 to confirm the functionality of ERE2. The luciferase reporter gene activity assay showed that knockdown of ERα expression decreased luciferase activity in the WT reporter gene transfection group, while there was no significant change in the Mut group. Conversely, overexpression of ERα significantly increased luciferase activity only in the WT reporter-transfected group (Fig. 4E, F).

Together, the results presented in Fig. 4A-F demonstrate that ERα can transcriptionally regulate CDH5 expression in BLCA cells.

### Mechanism dissection of how infiltrated macrophages increase ERα expression in BLCA cells: *via* IL-17 A-mediated epigenetic mechanism

**Fig. 5:**
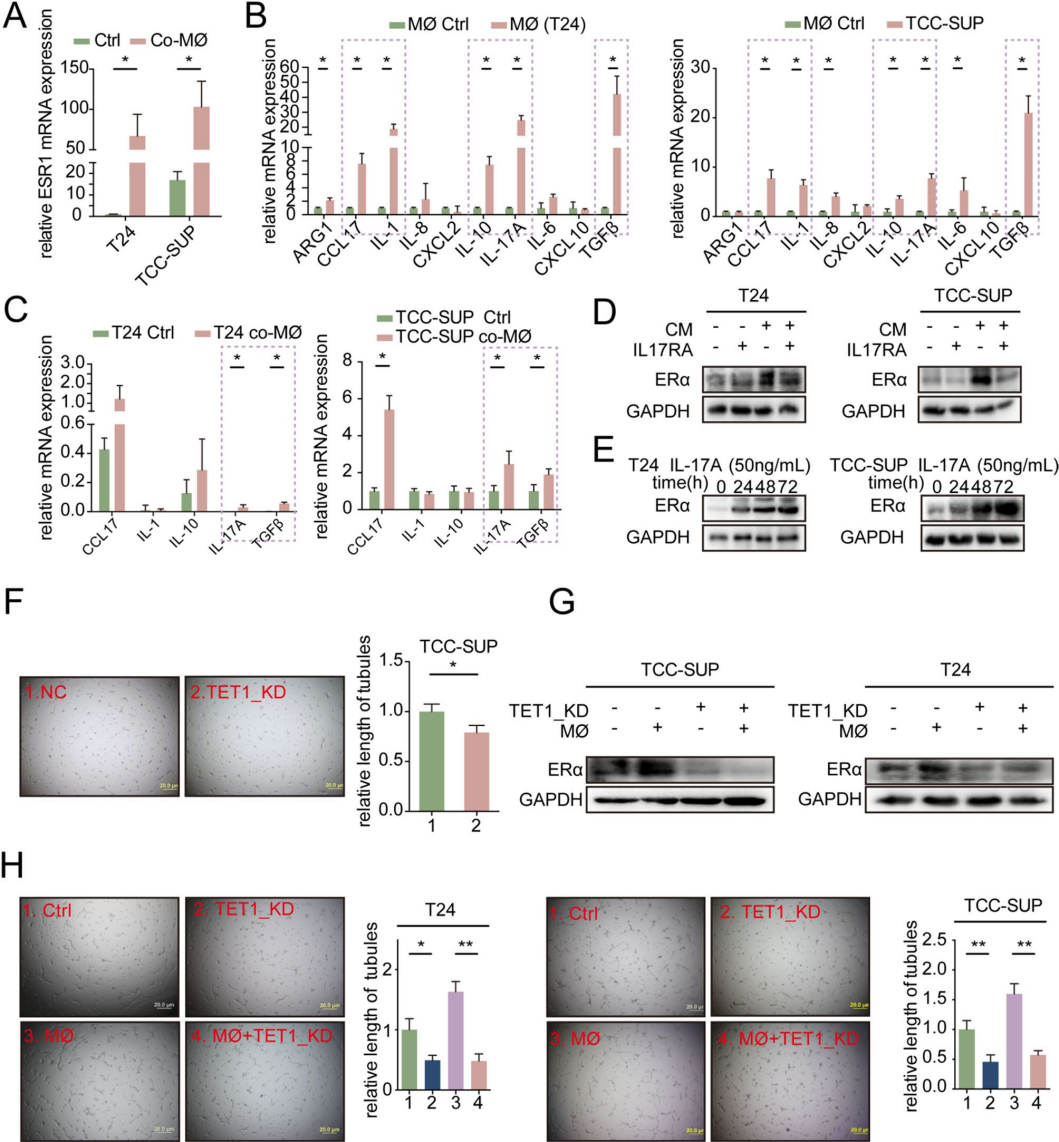
Macrophages increase ERα expression in BLCA cells *via* IL-17 A-mediated epigenetic mechanism. (**A**) qRT-PCR assay for detecting ERα gene (ESR1) expression in T24 and TCC-SUP cells co-cultured with macrophages versus blank control. (**B**) qRT-PCR assay was used to test the cytokines and chemokines secreted by macrophages in macrophages co-cultured with T24 cells (left) versus blank control and in macrophages co-cultured with TCC-SUP cells (right) versus blank control. (**C**) Analysis of cytokines screened from Fig. 5B in T24 cells (left panel) and TCC-SUP cells (right panel) co-cultured with macrophage versus blank control. (**D**) ERα expression in T24 cells (left panel) and TCC-SUP (right panel) cells treated as indicated. (**E**) ERα expression in T24 cells (upper panel) and TCC-SUP cells (lower panel) treated with IL-17 A at 50ng/ml concentration at the different indicated times. (**F**) 2D VM formation assay was performed in TCC-SUP cells transfected with NC and TET1-KD virus. (**G**) ERα expression in T24 cells (left panel) and TCC-SUP cells (right panel) treated as indicated. (**H**) 2D VM formation assay was performed in T24 cells (upper panel) and TCC-SUP cells (lower panel) treated as indicated. *For F and H*, quantitation is shown in the graph at right. Data are presented as means ± SD. *p < 0.05, **p < 0.01,* ns = no significance compared with the control*

Numerous research indicates that cytokines secreted by macrophages can influence the biological functions of tumors [11, 27]. Our qRT-PCR analysis revealed a significant increase in ESR1 mRNA expression in T24 and TCC-SUP cells following co-culture with macrophages, suggesting that the upregulation of ERα may be controlled at the transcriptional level (Fig. 5A). We screened ten potential cytokines produced by macrophages in co-culture settings with or without BLCA cells (Fig. 5B). Subsequently, we examined these five elevated cytokines in T24 and TCC-SUP cells with or without co-culture with macrophages, ultimately identifying IL-17 A and TGFβ as promising candidates (Fig. 5C). Notably, a related study on cytokine regulation of ERα expression in endometrial cancer cells highlighted that IL-17 A could enhance ERα expression by stimulating ESR1 transcription [17]. This study also proposed that IL-17 A-induced ERα expression might be linked to TET1-mediated demethylation of the ESR1 gene promoter. In addition, our analysis of ESR1 promoter methylation levels using UALCAN (http://ualcan.path.uab.edu/) unveiled a significant reduction in ESR1 promoter methylation levels in BLCA tumors [28] (Sfig.4 A). Moreover, we assessed ESR1 promoter methylation levels in different clinical stages within the TCGA dataset, revealing substantial decreases in methylation levels in advanced stages (II, III, and IV) compared to stage I (Sfig.4B). Considering the above findings and our previous results, we propose the following hypothesis: macrophages might elevate ERα expression in BLCA cells through an epigenetic mechanism that involves IL-17 A-mediated TET1-dependent demethylation of the ESR1 promoter [29, 30].

Based on the above hypothesis, we sought to verify the regulatory role of IL-17 A in ERα expression by introducing an IL-17 A receptor antibody (IL17RA) into the co-culture system to block IL-17 A function. The results demonstrated that inhibiting IL-17 A function attenuated the macrophage-mediated upregulation of ERα expression in T24 and TCC-SUP cells (Fig. 5D). Subsequently, western blot assays confirmed that the addition of exogenous IL-17 A led to a time- and dose-dependent increase in ERα expression (Fig. 5E, Sfig.4 C). Based on the proposed mechanism, IL-17 A is believed to impact the demethylation of the ESR1 promoter through the TET1-mediated 5-hydroxymethylation pathway, consequently upregulating ERα expression. In order to exclude the direct regulation of CDH5 by IL-17 A and prove that IL-17 A in BLCA promotes CDH-5-dependent VM formation by specifically upregulating the expression of ERα, we added exogenous IL-17 A to ERα knockdown cells and detect the expression of CDH5. Results showed that knockdown of ERα expression reduces CDH5 expression with/without addition with exogenous IL-17 A (Sfig. 4D).

To investigate this further, we examined the expression of ERα in BLCA cells after knockdown of TET1 expression. Notably, TET1 knockdown significantly inhibited ERα expression and impaired VM formation (Fig. 5F, Sfig. 4E). Furthermore, TET1 knockdown in BLCA cells reversed the macrophage-induced upregulation of ERα expression and VM formation (Fig. 5G-H). We assessed the prognostic value of TET1 in BLCA using GEPIA [31], revealing that patients with high TET1 expression exhibited inferior survival, thereby providing additional evidence supporting its pro-tumor role in BLCA (Sfig. 4 F).

### Tumor-derived exosomes regulated by ERα from BLCA cells promote M2 polarization and IL-17 A production *via* the pten/pakt pathway

**Fig. 6:**
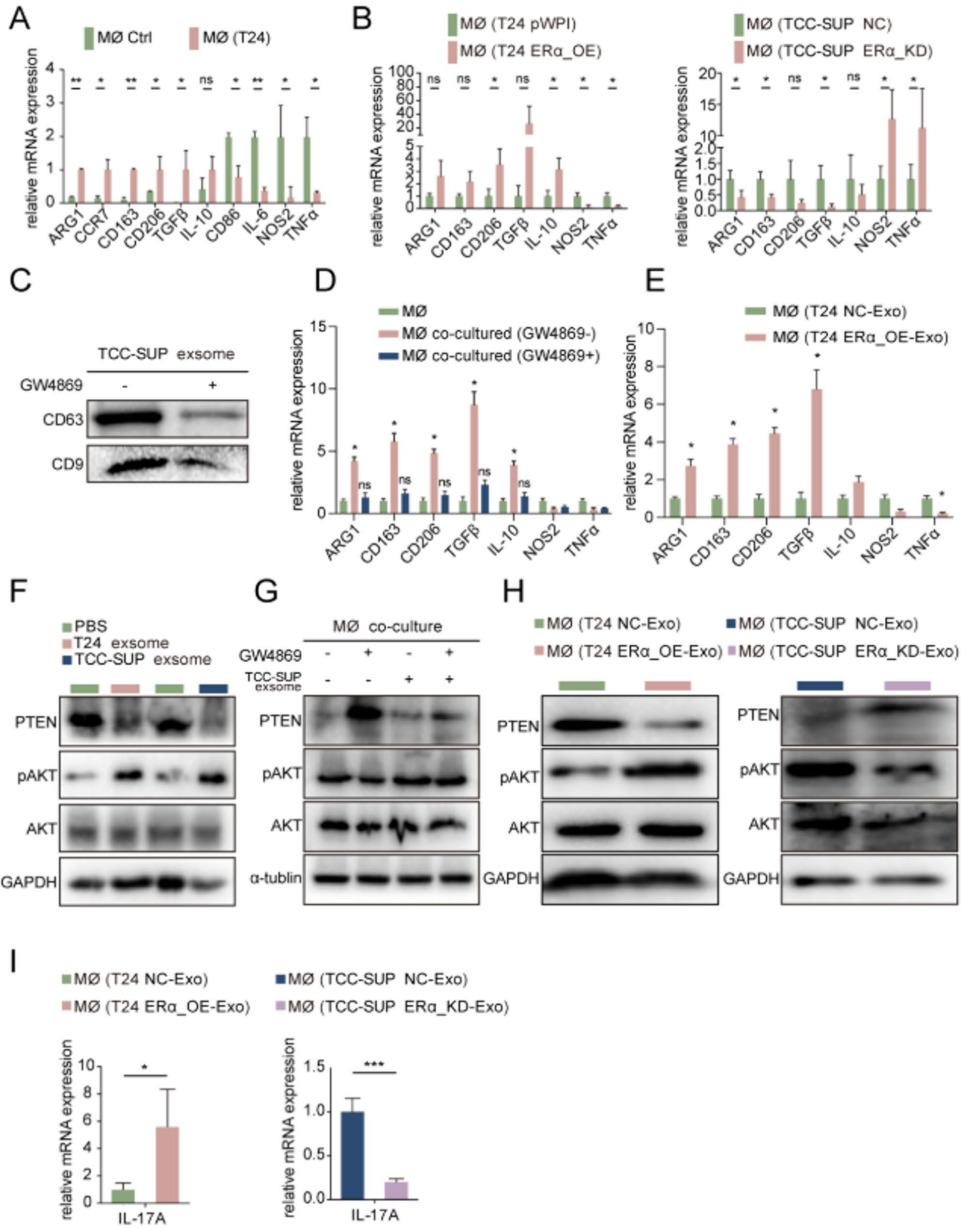
Tumor-derived exosomes regulated by ERα from BLCA cells can in turn promote M2 polarization and IL-17 A production via PTEN/pAKT pathway. (**A**) qRT-PCR for detection of the 11 M1/M2 macrophage markers in THP1 and co-cultured macrophages. (**B**) qRT-PCR for detection of the 7 selected M1 and M2 macrophage markers in macrophages co-cultured with T24 NC cells and T24 ER-OE cells (left), and in macrophages co-cultured with TCC-SUP-NC and TCC-SUP ERα-KD (right). (**C**) Western blot assay for detecting exosome markers CD63 and CD9 in exosomes collected from TCC-SUP treated with GW4869 (exosome-secreting inhibitor) versus blank control. (**F**) Western blot assay for PTEN, pAKT and AKT expression in macrophages treated with exosomes collected from T24 and TCC-SUP cells with PBS as control. (**G**) Western blot assay for PTE, pAKT and AKT expression in co-cultured macrophages treated as indicated. (**H**) Western blot assay for PTEN, pAKT and AKT expression in macrophages treated with exosomes collected from T24 NC cells and T24 ERα-OE cells (left panel) and macrophages treated with exosomes collected from TCC-SUP NC cells and TCC-SUP ERα-KD cells (right panel). (**I**) qRT-PCR assay for detection of IL-17 A expression in macrophages co-cultured with T24 NC cells and T24 ERα-OE cells (left) and macrophages co-cultured with TCC-SUP NC cells and TCC-SUP ERα-KD cells (right). Data are presented as means ± SD. *p < 0.05, **p < 0.01,* ns = no significance compared with the control*

TAMs play a key role in the inflammatory response and tumor microenvironment. The dual role of TAMs in BLCA depends on different polarization states classified as M1/M2 [11]. The interaction between macrophages and tumor cells is known to reshape the tumor microenvironment and promote tumor progression [32]. TAMs, particularly M2 type, are known to exert tumor-promoting functions within this microenvironment. According to the results in Fig. 1, it was found that the expression of ERα was positively correlated with the degree of infiltration of M2 macrophages. We thus hypothesized whether ERα is involved in regulating the M2 polarization of macrophages. To further test the hypothesis, we examined the expression of M1 and M2 markers in macrophages after coculture with or without BLCA cells. The results demonstrated a significant increase in most M2 markers following co-culture, while the levels of M1 markers were decreased (Fig. 6A). Subsequently, we detected the expression of M2 markers in macrophages after co-culture with T24 cells transfected with/without ERα-overexpression virus and TCC-SUP cells transfected with/without ERα-knockdown virus. As expected, the results showed that M2 markers were significantly increased in the T24 ERα-OE co-culture group, while M2 markers were significantly decreased in the TCC-SUP ERα-KD co-culture group, indicating that altering ERα expression in BLCA cells can effectively influence M2 macrophage polarization (Fig. 6B). According to previous studies, tumor-derived extracellular vesicles (EVs) containing small molecules such as microRNAs can mediate M2 macrophage polarization [33, 34]. To determine the involvement of tumor-derived exosomes in M2 macrophage polarization, the exosome inhibitor GW4869 was used to block the function of exosomes in co-culture system. The protein levels of exosome markers CD63 and CD9 were assessed using western blot, and the morphology of tumor cells-derived exosomes was examined by electron microscopy, all of which confirmed the presence of the exosomes we collected (Fig. 6C, Sfig.5 A). Results showed that the addition of GW4869 in the co-culture system could significantly block M2 polarization of macrophages (Fig. 6D) and the addition of exosomes from T24 ERα-OE cells significantly induced M2 macrophage polarization (Fig. 6E).

Given the feedback regulation of M2 macrophage polarization by ERα expression in BLCA cells, we investigated the potential underlying mechanisms. Numerous studies have implicated pAKT-mediated signaling pathways in the regulation of M2 polarization [33, 35]. Interestingly, the PTEN/pAKT axis has also been reported to regulate IL17 expression in macrophages [36]. To verify the involvement of the PTEN/pAKT axis in promoting M2 macrophage polarization, we examined the expression of PTEN and pAKT in macrophages treated with T24-CM and TCC-SUP-CM. The results showed that the addition of CM from both T24 and TCC-SUP decreased the expression of PTEN and increased the expression of pAKT in macrophages (Sfig. 5B). In addition, we found that T24 and TCC-SUP cells-derived exosomes can affect the PTEN/pAKT pathway in macrophages (Fig. 6F). The addition of GW4869 to the co-culture system deprived tumor cells of their ability to regulate the PTEN/pAKT pathway in macrophages, whereas providing exosomes reversed this deprivation (Fig. 6G). Furthermore, we assessed the expression of PTEN and pAKT in macrophages which were treated with exosomes collected from T24 ERα-OE cells and TCC-SUP ERα-KD cells. The results showed that PTEN expression was increased after the addition of T24 ERα-OE cells-derived exosomes and decreased after the addition of exosomes from TCC-SUP ERα-KD cells, while the expression of pAKT was changed opposite to that of PTEN (Fig. 6H). The mRNA expression of IL-17 A in macrophages exhibited changes consistent with the alteration of ERα expression in TCC-SUP and T24 cells, aligning with the reported transcriptional regulation of IL-17 A by the PTEN/pAKT pathway [36] (Fig. 6I). Also, KEGG pathway analysis revealed that PTEN negatively regulated IL17A production in BLCA (Sfig.5 C).

Taken together, the results in Fig. 6A-I suggest that ERα in BLCA cells can promote M2 polarization and IL-17 A production by regulating the PTEN/pAKT pathway in macrophages through exosomes.

### Mechanism dissection of ERα down-regulation of pten/pakt pathway: direct targeting of 3’UTR of PTEN mRNA by miR-642a-5p

**Fig. 7:**
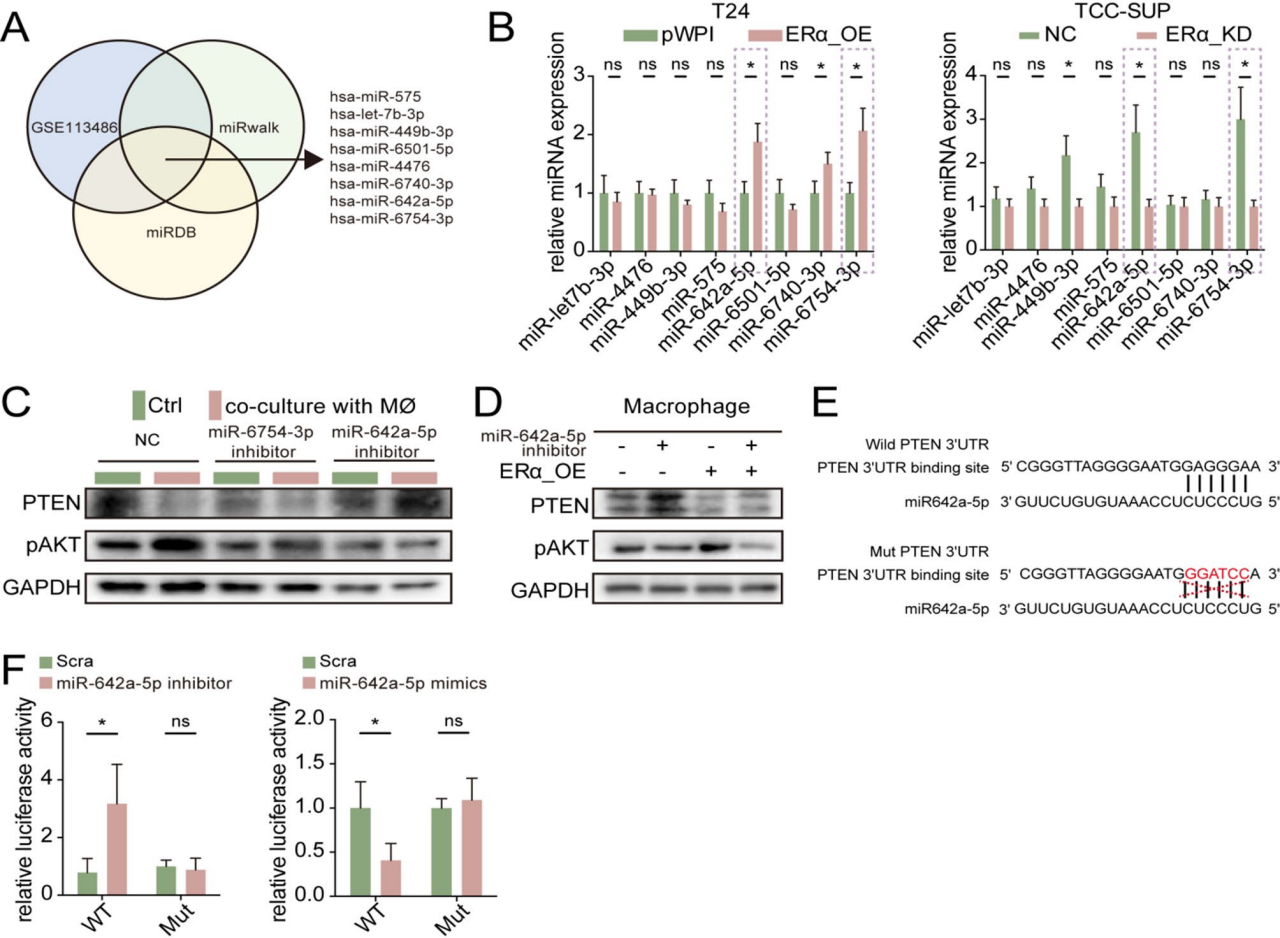
ERα-regulated miR-642a-5p down-regulate PTEN/pAKT pathway by targeting of 3’UTR of PTEN mRNA. (**A**) Bioinformatics analysis of a set of potential miRNAs predicted to regulate PTEN in BLCA. (**B**) qRT-PCR assay for the 8 miRNAs expression in T24 cells transfected with NC or ERα-OE (left) and in TCC-SUP cells transfected withNC or ERα-KD (right). (**C**) Western blot assay for detection of PTEN and pAKT expression in macrophages with/without co-cultured T24 cells transfected with pLKO-vector, miR-6754-3p inhibitor and miR-642a-5p inhibitor. (**D**) Western blot assay for PTEN and pAKT expression in macrophages co-cultured with T24 cells transfected as indicated. (**E**) The structure of WT or Mut binding sites between PTEN 3’UTR and miR-642a-5p. (**F**) Luciferase reporter activity was measured and analyzed in THP-1 cells transfected with pLKO/ miR-642a-5p inhibitor (left) and THP-1 cells transfected with pLKO/oemiR-642a-5p (right) after addition of WT or Mut 3’UTR of PTEN. Data are presented as means ± SD. *p < 0.05, **p < 0.01,* ns = no significance compared with the control*

We observed that exosomes derived from BLCA cells promote M2 polarization and IL-17 A production in macrophages by modulating the PTEN/pAKT signaling pathway. We also found that altering ERα expression in BLCA cells did not change the expression of PTEN at mRNA levels in macrophages (Sfig.5D). As PTEN may be regulated at post-transcriptional level, we focused on miRNAs, well-known for their encapsulation in exosomes and their role in post-transcriptional regulation. Using bioinformatic analysis, we screened eight candidate miRNAs involved in PTEN regulation in BLCA (Fig. 7A), and their expression was assessed by modulating ERα expression in BLCA cells, following which we identified miR-642a-5p and miR-5754-3p as promising candidates (Fig. 7B). To confirm the functions of these candidates, we co-cultured T24 cells with macrophages following transfection with vector-based inhibitors targeting miR-642a-5p and miR-5754-3p. Subsequently, we evaluated PTEN/pAKT expression in macrophages. Intriguingly, macrophages co-cultured with T24 cells transfected with the miR-642a-5p inhibitor exhibited significant alterations in the PTEN/pAKT signaling axis compared to those co-cultured with T24-pLKO cells (Fig. 7C). Moreover, inhibition of miR-642a-5p in BLCA cells effectively reversed the influence of ERα on PTEN/pAKT in macrophages (Fig. 7D).

Based on the aforementioned findings, we conducted a luciferase reporter assay to provide further confirmation that miR-642a-5p post-transcriptionally downregulates PTEN expression by binding to its 3’UTR. First, we identified potential miR-642a-5p targeting sites within the PTEN 3’UTR using starBase v2.0 [37]. Subsequently, we utilized the psiCHECK2 vector to construct both wild-type (WT) and mutant-type (Mut) 3’UTR versions, with the latter lacking the miR-642a-5p target site (Fig. 7E). The luciferase assay results demonstrated that the luciferase activity in THP-1 cells transfected with the wild-type PTEN 3’UTR was significantly increased upon miR-642-5p inhibitor addition and decreased when miR-642-5p was overexpressed. Conversely, luciferase activity remained largely unchanged in THP-1 cells transfected with the mutant PTEN 3’UTR (Fig. 7F).

Given that the expression of miR-642a-5p in BLCA cells is influenced by ERα (Fig. 7B), we conducted a CHIP assay to investigate whether ERα could transcriptionally regulate miR-642a-5p expression by binding to its promoter. We also examined three predicted estrogen response elements (EREs) located within the promoter region of miR-642a-5p that could potentially bind to ERα. The results suggest that ERE1 may be the most promising candidate (Sfig.6 A-B). Subsequently, we performed a luciferase reporter assay to verify the functionality of ERE1 using pGL3 reporter plasmids containing either the wild-type (WT) or mutant (Mut) ERE1 (Sfig.6 C). The luciferase assay results demonstrated a significant increase in luciferase activity in the WT reporter gene transfection group upon ERα overexpression, whereas it sharply decreased following ERα knockdown. In contrast, there was no change observed in the Mut group (Sfig. 6D).

### Preclinical study using an in vivo mouse model confirming that targeting ERα represses tumor progression by impeding M2 macrophage polarization and VM formation

**Fig. 8:**
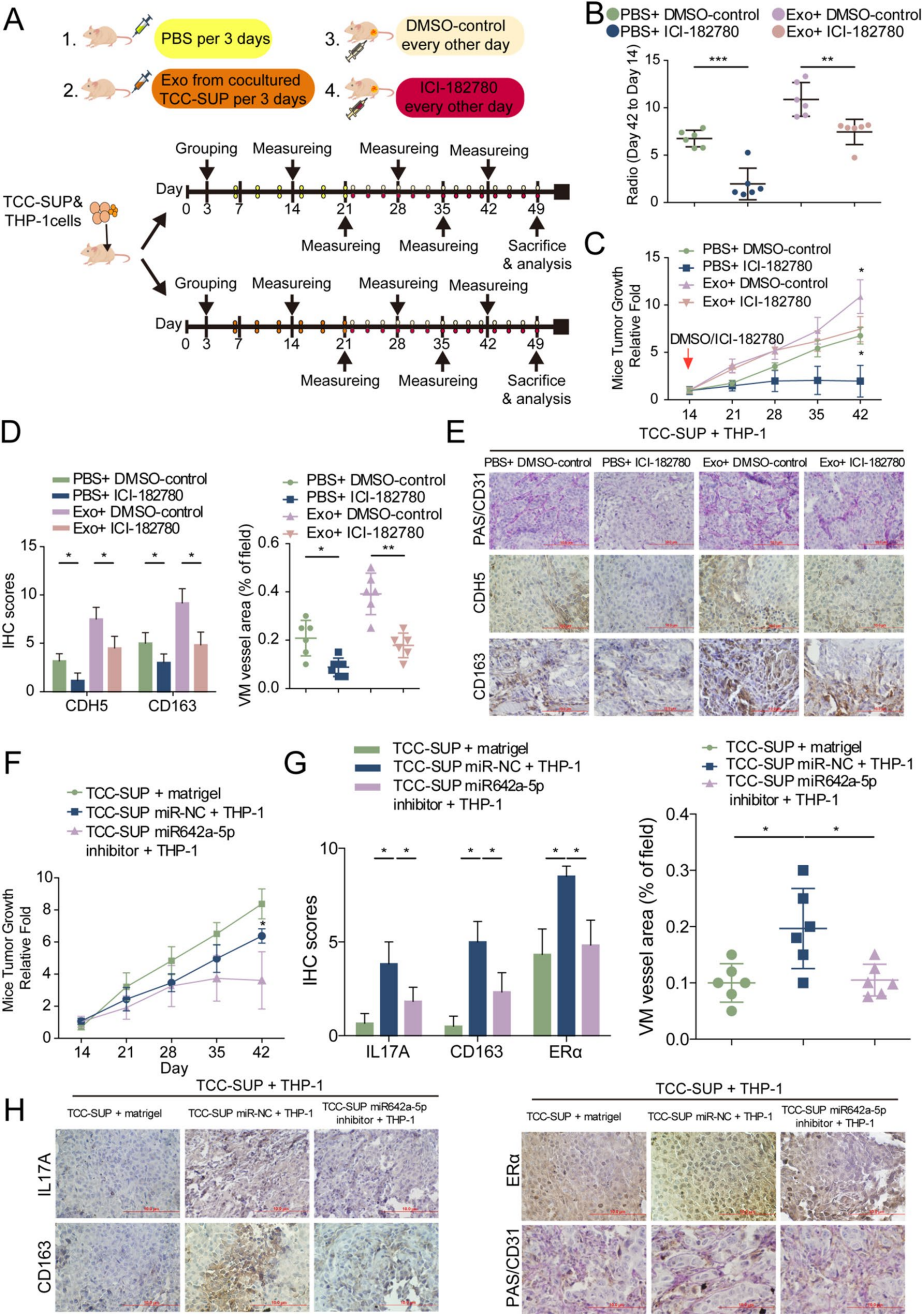
Targeting ERα represses tumor progression through impeding M2 macrophage polarization and VM formation in vivo.(**A**) Diagram of the in vivo study design. (**B**) Quantification for day 42 tumor growth ratio. (**C**) Tumor growth curve of mice from the four groups. (**D**) Quantification of the relative IHC staining intensity for CDH5 and CD163 expression (left) and evaluation of area percentage of VM vessels (right). (**E**) Representative images of IHC staining for VM vessel area (PAS+/CD31 − tumor cell-dependent vessels indicated by red triangle), CDH5 and CD163 in implanted tumor tissues from mice received indicated treatment. (**F**) Tumor growth curve after 42 days in mice implanted with TCC-SUP + Matrigel, TCC-SUP-pLKO + THP-1, TCC-SUP-miR642a-5p inhibitor + THP-1. (**G**) Quantification of the relative IHC staining intensity for IL-17 A, CD163 and ERα expression (left) and evaluation of area percentage of VM vessels (right). (**H**) IHC staining for IL-17 A, CD163, ERα and VM vessel area (PAS+/CD31 − tumor cell-dependent vessels indicated by red triangle) in implanted tumor tissues from indicated groups. Data are presented as means ± SD. *p < 0.05, **p < 0.01, ns = no significance compared with the control

To validate the above findings, we constructed an in vivo xenograft mouse model. TCC-SUP cells and THP-1 cells were co-injected subcutaneously into the flanks of mice, and tumor-bearing mice were treated with exosomes from co-cultured TCC-SUP cells or PBS as control via tail-vein every 3 days for two weeks. To target ERα in tumors of the above mice model, its antagonist ICI-182,780 or DMSO was injected intraperitoneally every other day for 4 weeks until the mice reached the treatment endpoints (Fig. 8A). According to different treatments, the mice were divided into four groups: PBS + DMSO group, PBS + ICI-182,780 group, exosomes + DMSO group and exosomes + ICI-182,780 group (6 in each group). Tumor growth rates and tumor growth curves in each group showed that exosomes from co-cultured BLCA cells significantly promoted tumor growth, and inhibiting ERα by ICI-182,780 could reduce tumor growth (Fig. 8B, C). The results of IHC staining analysis confirmed that tumor cell-derived exosomes promoted VM formation by upregulating CDH-5 expression and promoted M2 polarization of TAMs. Furthermore, inhibition of ERα by adding ICI-182,780 significantly reduced CDH5-regulated VM formation and M2 polarization in a BLCA mice model constructed with TCC-SUP and THP-1 systems (Fig. 8D, E).

To further investigate whether the role of miR-642a-5p in vivo corresponds with our in vitro findings, we conducted another xenograft animal experiment. In this experiment, we injected three different combinations into the flanks of mice: TCC-SUP cells alone, TCC-SUP cells transfected with pLKO vector + THP-1 cells, and TCC-SUP cells transfected with pLKO-miR-642a-5p inhibitor + THP-1 cells. After six weeks, we observed that co-injection of TCC-SUP-pLKO + THP1 cells resulted in significantly larger tumors compared to those produced by TCC-SUP cells or TCC-SUP-pLKO-miR-642a-5p-inhibitor + THP-1 cells. Importantly, co-injection of TCC-SUP-pLKO-miR-642a-5p inhibitor + THP-1 cells led to a significant inhibition of tumor growth compared to the other two groups (Fig. 8F). Furthermore, IHC staining revealed that the inhibition of miR-642a-5p reduced macrophage-induced IL-17 A expression and M2 polarization, consequently reducing ERα-regulated VM formation in vivo (Fig. 8G-H).

**Fig. 9:**
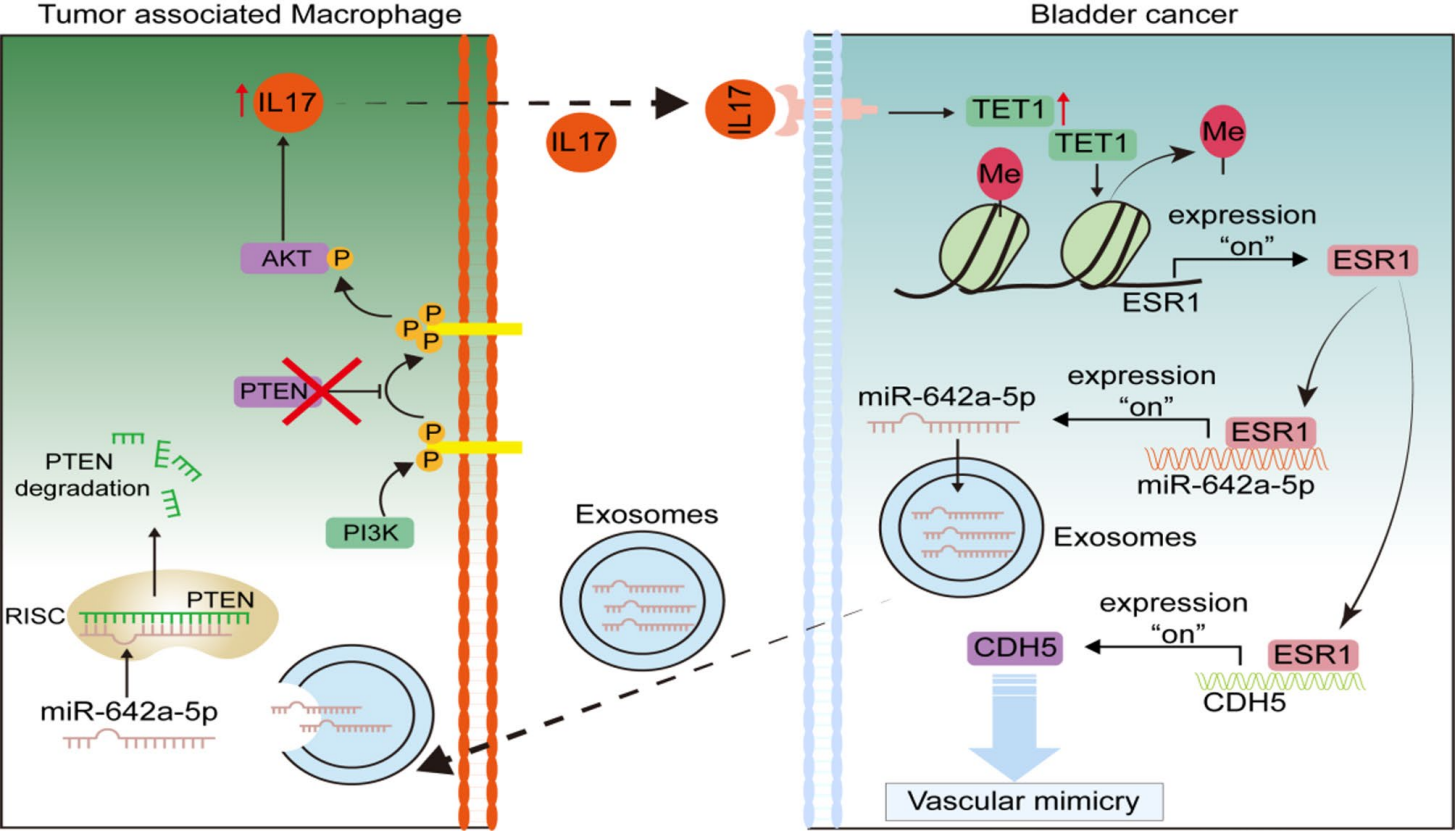
Schematic illustration of the feedback circuit between macrophages and cancer cells in BLCA microenvironment

Taken together, these results demonstrate that macrophages enhance tumor growth and vascular mimicry (VM) formation by inducing the upregulation of ERα expression in BLCA. Conversely, ERα promotes M2 polarization and increases IL-17 A expression in macrophages by upregulating miR-642a-5p, which is shuttled in exosomes from BLCA cells. In addition, this study suggests an ERα-dependent feedback loop operating between macrophages and cancer cells within the BLCA microenvironment, significantly contributing to the promotion of BLCA progression (Fig. 9).

## Discussion

While BLCA is more prevalent in men than women, it is noteworthy that women tend to progress to advanced stages and experience worse overall survival [38]. ERα, a well-known estrogen receptor, has been traditionally considered to exert a tumor-suppressive role in the initiation phase of BLCA. However, its precise role in the progression phase has remained elusive [5, 39]. Interestingly, an analysis of the TCGA database revealed a significant difference in the prognostic value of ERα in early and late-stage BLCA patients. In advanced BLCA cases, elevated ERα expression levels were associated with poor survival outcomes, while in early-stage patients, higher ERα expression correlated with a more favorable prognosis, thereby supporting our hypothesis that ERα may indeed play a tumor-promoting role in the progression of BLCA.

VM is a phenomenon observed in various malignant tumors, including lung cancer, hepatocellular carcinoma, melanoma, gastric cancer, renal carcinoma, and BLCA [40, 41]. Clinical analysis of 15 different cancer types revealed that patients with VM-positive tumors tend to exhibit significantly lower 5-year overall survival rates, indicating that VM formation is associated with a poor prognosis for cancer patients [42]. VM is characterized by its independence from epithelial cells and is viewed as a potential target for anti-angiogenic therapy [43, 44]. Therefore, targeting the regulatory pathways underlying VM formation in BLCA represents a promising strategy for enhancing combination therapies. Our research provides a novel perspective by highlighting that macrophage-induced upregulation of ERα expression promotes VM formation in BLCA, both in vitro and in vivo. Beyond its role in enhancing VM formation, ERα also exerts influence over M2 macrophage polarization through the upregulation of the exosomal miR-642a-3p/PTEN/pAKT pathway, thereby fostering an immunosuppressive microenvironment. The activated PTEN/pAKT pathway in macrophages, in turn, further increases IL-17 A expression by stimulating its downstream transcription factor NF-κB, consequently promoting tumor progression through a feedback loop.

This study briefly elucidated the mechanism by which macrophages upregulate ESR1 transcription. Prior research documented that the cytokine IL-17 A, produced by macrophages, can stimulate ERα expression by facilitating TET1-mediated hydroxymethylation of the ESR1 gene promoter [17]. The TET family of enzymes is responsible for catalyzing hydroxymethylation, a process that involves converting 5-methylcytosine (5-mC) into 5-hydroxymethylcytosine (5-hmC), which serves as an intermediate step in the DNA demethylation process [45, 46]. Data from the TCGA database corroborated our findings, indicating that the methylation levels of the ESR1 promoter were significantly lower in primary BLCA tumors compared to normal tissues. Furthermore, ESR1 promoter methylation levels exhibited a significant decrease in late-stage patients compared to their early-stage counterparts. Taken together, this evidence supports our conclusion that the macrophage-induced upregulation of ERα in BLCA relies on an IL-17 A-mediated epigenetic mechanism.

Previous studies have highlighted the pivotal role of tumor cells in reshaping the tumor microenvironment to promote cancer progression, primarily through the release of exosomes [47, 48]. Tumor cell-derived exosomes have been shown to transmit miRNAs, which play critical roles in processes such as M2 macrophage polarization and recruitment of TAMs, which influence tumor proliferation, vascularization and metastasis [49, 50]. Here, we found that ERα upregulates tumor-derived miR-642a-3p transmitted through exosomes, affecting M2 polarization by altering the PTEN/pAKT signaling pathway in macrophages and reinforcing the unfavorable tumor immunosuppressive environment. The upregulation of ERα in bladder tumor cells can further promote VM formation and strengthen this positive regulation. ERα serves as a link between tumor-infiltrating macrophages and VM formation in BLCA. Macrophage infiltration and VM formation were significantly reduced in the BLCA tissue of ERα KO mice. In human clinical tissues, we found that ERα-positive cases tended to have both higher macrophage infiltration and VM formation. These findings serve as evidence to support our hypothesis and provide a basis for further exploring new perspectives in developing novel treatment strategies for BLCA.

In conclusion, macrophages-induced ERα in BLCA enhances VM formation by transcriptionally upregulating CDH5. The increased ERα expression then promotes M2 polarization via miR-642a-3p delivered by tumor-derived exosomes. Thus, targeting this newly identified signaling pathway could be a promising therapeutic strategy for the treatment of BLCA patients.

## Electronic supplementary material

Below is the link to the electronic supplementary material.

Supplementary Material 1

Supplementary Material 2

Supplementary Material 3

## Data Availability

No datasets were generated or analysed during the current study.
