# Supplementary material for: ERα-dependent crosstalk between macrophages and cancer cells potentiates vasculogenic mimicry and M2 macrophage polarization in bladder cancer
Source: Cell Commun Signal. 2025 Jul 15;23:339. doi: 10.1186/s12964-025-02297-7 (PMC12261844; doi:10.1186/s12964-025-02297-7)
Supplement: Supplementary file 1 — Supplementary Material 1 [file 12964_2025_2297_MOESM1_ESM.docx]

**Sfig. 1
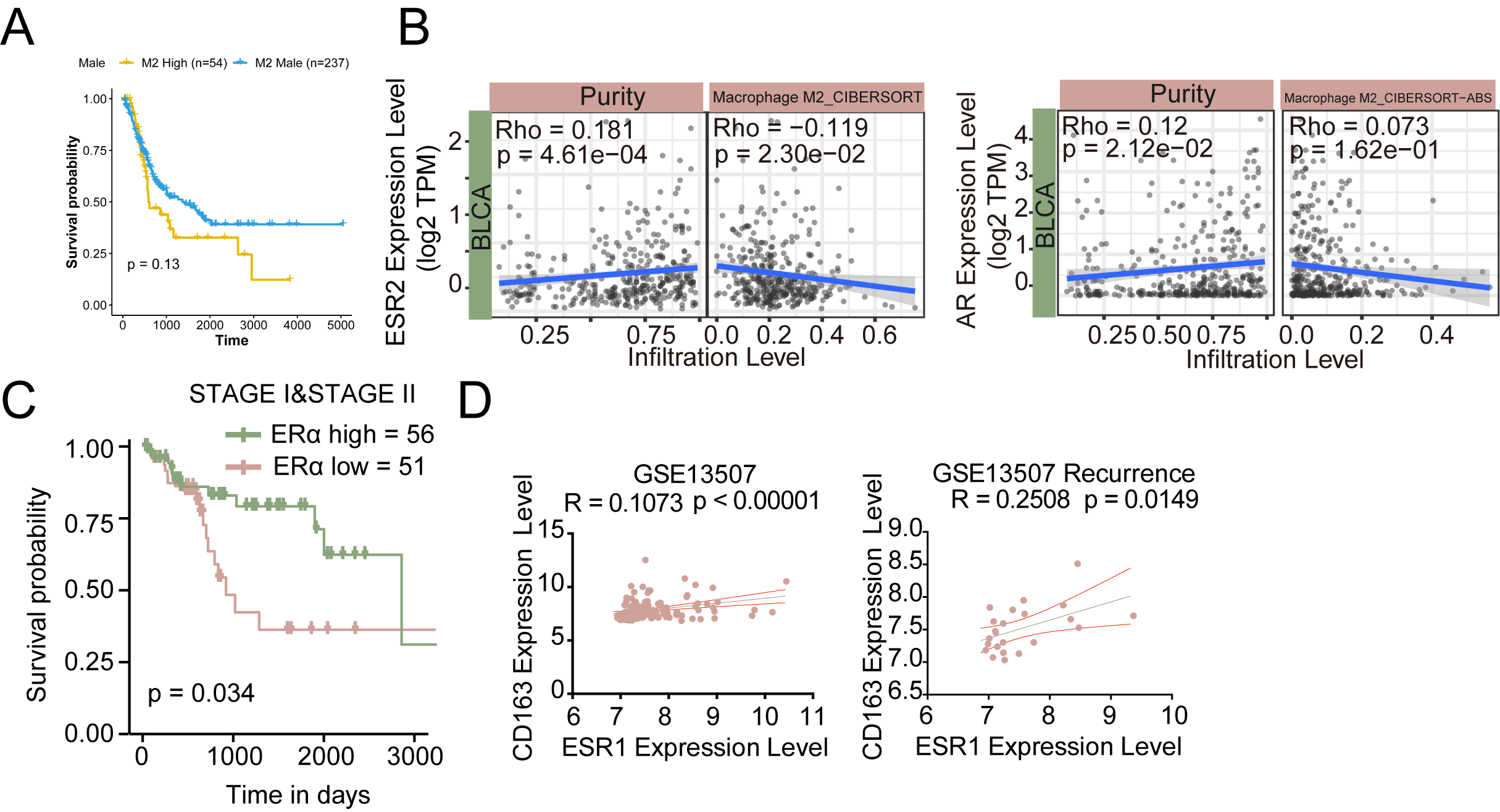
**

(A) Prognostic value of M2 macrophage infiltration level in male BLCA patients. (B) Correlation between ESR2 gene expression and the level of M2 macrophage infiltration (left); correlation between AR expression and M2 macrophage infiltration (right). (C) Prognostic value of ERα expression in BLCA patients at stage I and stage II. (D) Correlation of CD163 expression levels with ESR1 expression levels in primary tumors (Left) and recurrent tumors (Right) of BLCA patients using the GSE13507 dataset.

**Sfig. 2**

**
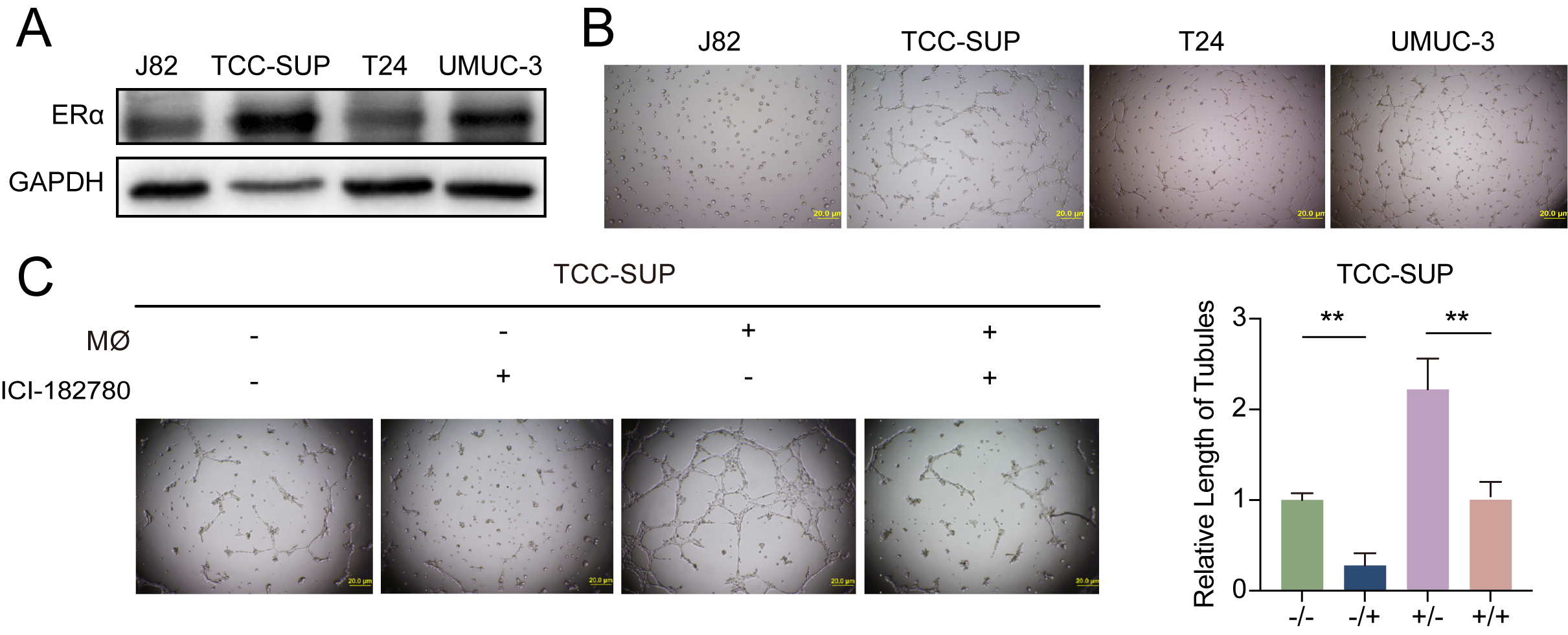
**

(A) Western blot assay for detection of ERα expression in four BLCA cell lines. (B) 2D VM formation assay was performed in the four BLCA cell lines. (C) 2D VM formation assay was performed in TCC-SUP cells treated as indicated. *For C, quantitation is shown in the graph at right. Data are presented as means ± SD. *p < 0.05, **p < 0.01, ns = no significance compared with the control.*

**Sfig. 3**

**
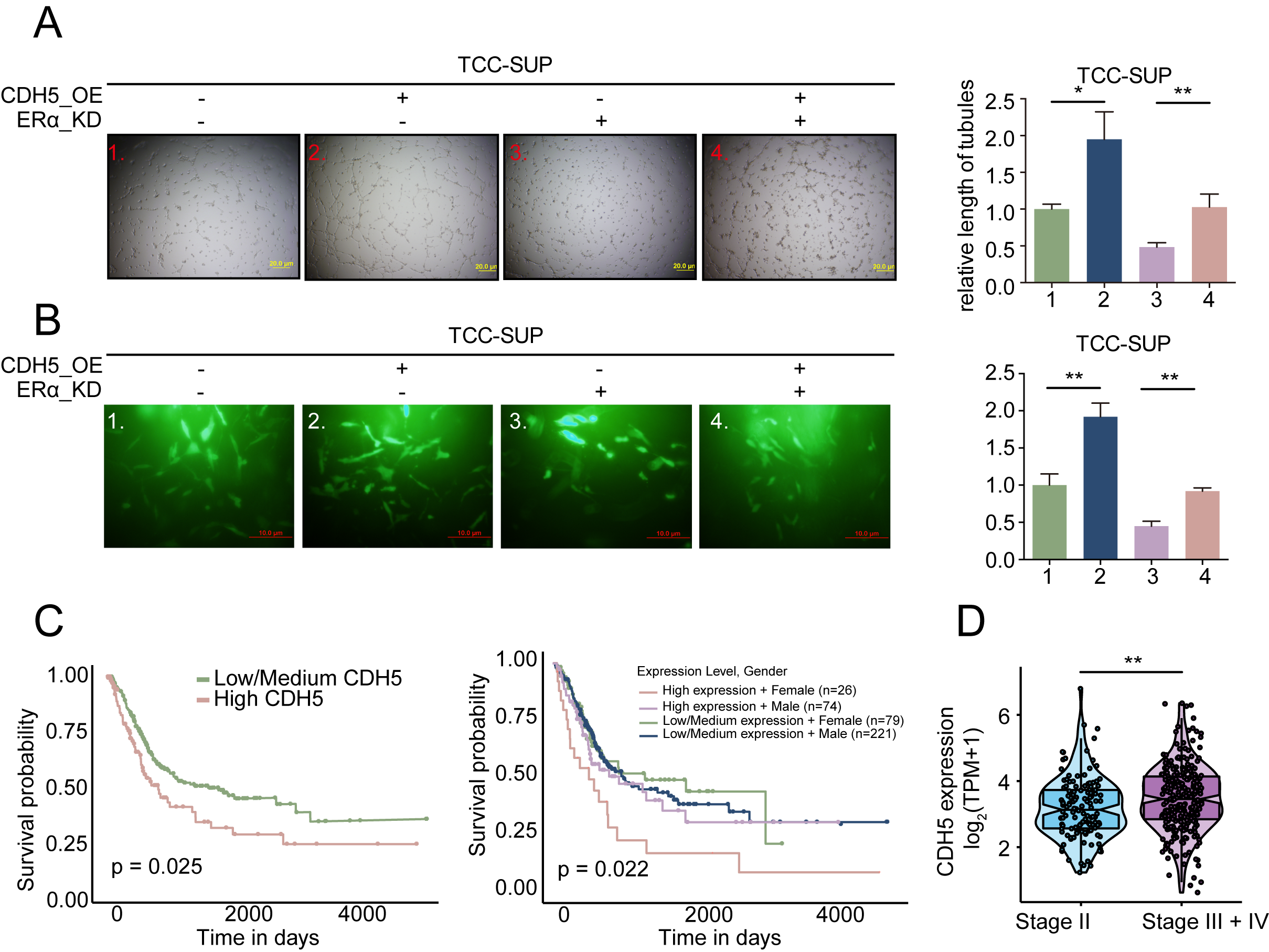
**

(A) 2D VM formation assay was performed in TCC-SUP cells transfected as indicated in the upper panel. (B) 3D VM formation assay was performed in TCC-SUP cells transfected as indicated in the upper panel. (C) Survival analysis of CDH5 gene expression in BLCA patients based on TCGA datasets. (D) Expression of CDH5 in stage II and stage III+IV BLCA patients based on TCGA datasets. *For A and B, quantitation is shown in the graph at right. Data are presented as means ± SD. *p < 0.05, **p < 0.01, ns = no significance compared with the control.*

**Sfig. 4**

**
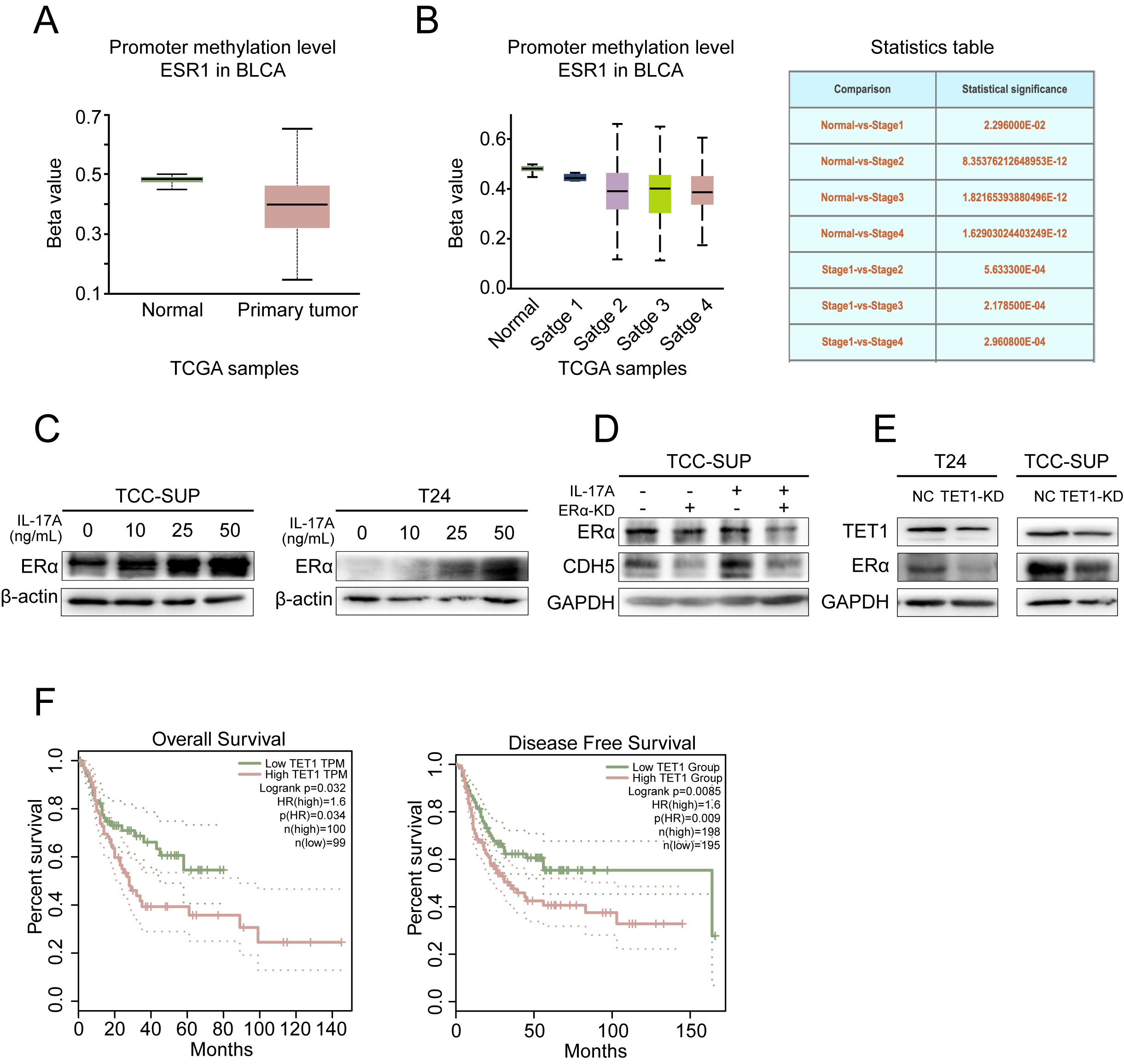
**

(A) The promoter methylation level of ESR1 in BLCA. (B) The promoter methylation level of ESR1 in TCGA samples of different stages of BLCA. (C) Western blot assay for detection of ERα expression in TCC-SUP cells (left) and T24 cells (right) treated with IL-17A at different concentration for 48h. (D) Western blot assay for CDH5 expression in TCC-SUP cells treated as indicated. (E) Western blot assay for TET1 and ERα expression in T24 cells (left panel) and TCC-SUP cells (right panel), both transfected with TET1 knockdown virus (TET1-KD) versus mock vector (NC). (F) Prognostic value of TET1 expression in BLCA patients.

**Sfig. 5**

**
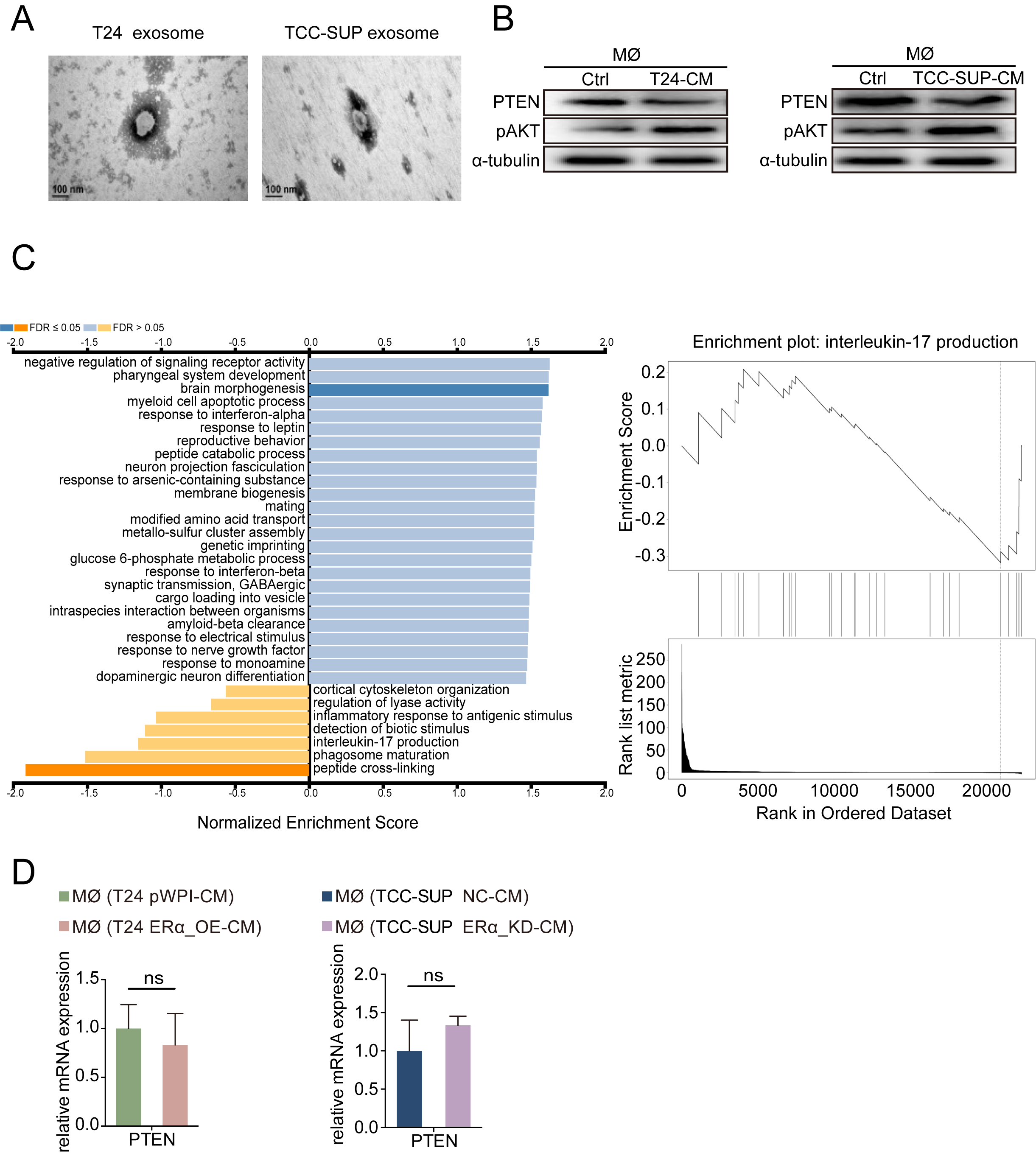
**

(A) Photographs of exosomes collected from T24 and TCC-SUP. (B) Western blot assay for PTEN and pAKT expression in macrophages treated with conditioned medium from T24 (left) and TCC-SUP (right). (C) Analyzing signaling pathways enriched by PTEN in BLCA by online tool LinkedOmics. (D) qRT-PCR assay for detection of PTEN expression in macrophages co-cultured with T24 NC cells and T24 ERα-OE cells (upper panel) and macrophages co-cultured with TCC-SUP NC cells and TCC-SUP ERα-KD cells (lower panel). *Data are presented as means ± SD. *p < 0.05, **p < 0.01, ns = no significance compared with the control.*

**Sfig. 6**

**
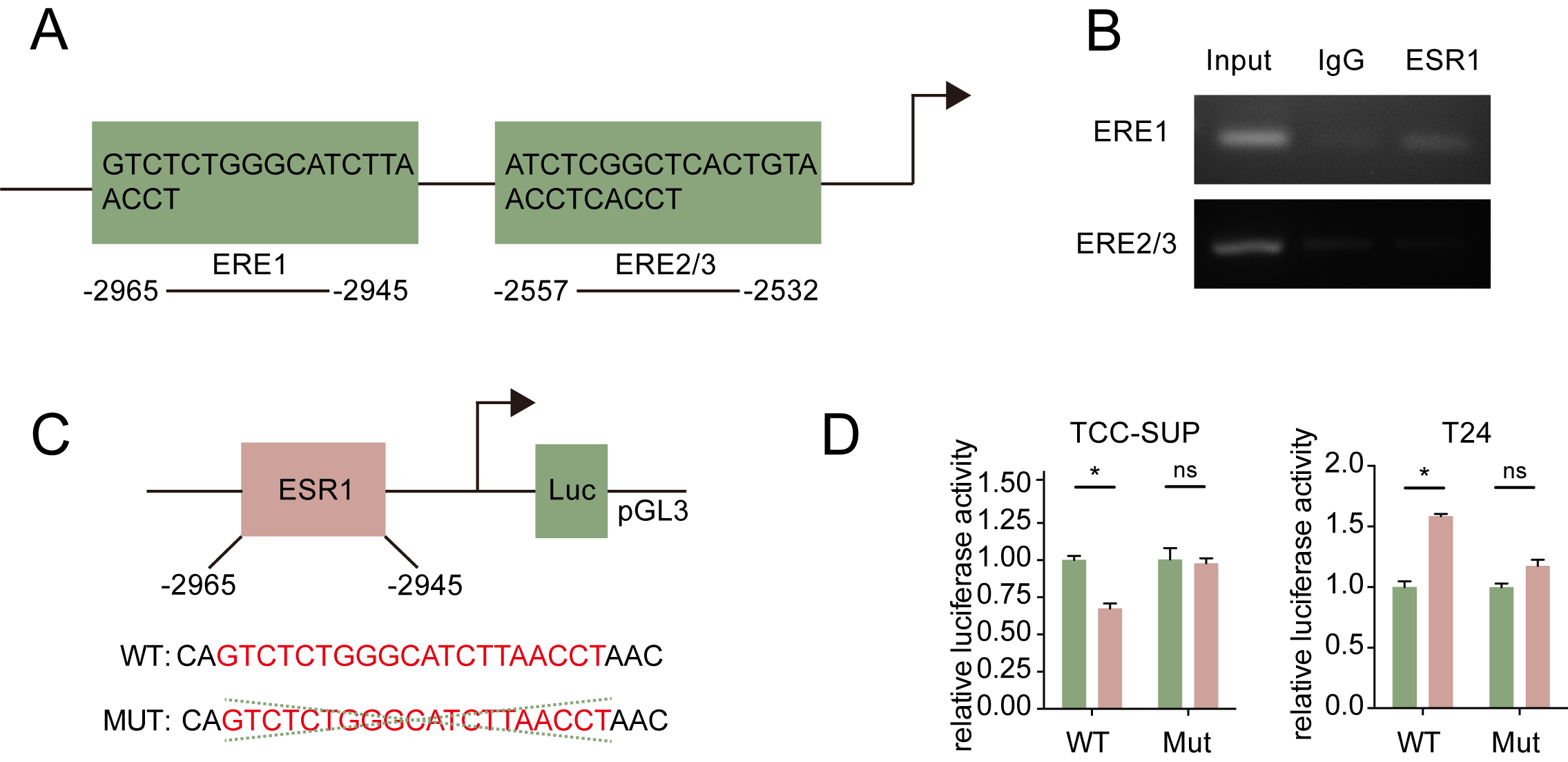
**

(A) The sketch structure of ERE binding sites in 3kb miR-642a-5p promoter region. (B) CHIP assay was performed to detect ERα binding on the proposed ERE1 on miR-642a-5p promoter region. (C) Illustrative diagram of WT and Mut pGL3-miR-642a-5p promoter-reporter constructs. (D) Luciferase activity of reporter plasmids carrying ERE-WT or ERE-Mut pGL3-miR-642a-5p promoter was measured and analyzed in T24 NC cells/ T24 ERα-OE (left) and TCC-SUP cells with pLKO/shERα (right). *Data are presented as means ± SD. *p < 0.05, **p < 0.01, ns = no significance compared with the control.*
