# Supplementary material for: ERα-dependent crosstalk between macrophages and cancer cells potentiates vasculogenic mimicry and M2 macrophage polarization in bladder cancer
Source: Cell Commun Signal. 2025 Jul 15;23:339. doi: 10.1186/s12964-025-02297-7 (PMC12261844; doi:10.1186/s12964-025-02297-7)
Supplement: Supplementary file 2 — Supplementary Material 2 [file 12964_2025_2297_MOESM2_ESM.docx]

Supplementary method

**Bioinformatics analysis:** We assessed the correlation between M2 macrophage infiltration and the prognosis of BLCA patients using the TCGA-BLCA cohort and acquired the level 3 gene expression profiles of 396 patients from the TCGA Data Portal (https://tcga-data.nci.nih.gov/tcga), along with clinicopathological data, including gender and tumor stage. To calculate the infiltration of M2 macrophages, we utilized CIBERSORT, an algorithm capable of quantifying the proportion of Tumor-Infiltrating Immune Cells (TIICs) based on 547 signature genes [21]. Furthermore, we examined the association between ESR1 and CDH5 using the Pearson correlation method across 34 different tumor types. The original expression data were obtained from the UCSC Xena platform (https://xenabrowser.net).

**Immunohistochemistry (IHC) staining:** The procedure was performed according to the routine manual created by our team previously [22]. Following tissue sample preparation, fixation, deparaffinization, hydration, antigen retrieval and blocking, the tissue slices were incubated overnight with primary antibodies in a 3% BSA solution dissolved in PBS at 4°C. Subsequently, biotinylated secondary antibodies (Vector Laboratories, Burlingame, CA, USA) were applied, and visualization was achieved using the VECTASTAIN ABC peroxidase system and the 3,3'-diaminobenzidine (DAB) kit (Vector Laboratories, Burlingame, CA, USA). The percentage of positively stained cells was quantified per high-power field (HPF) at 400× magnification. The primary antibodies used included anti-CD163 (M2 macrophage marker; Thermo Fisher Scientific, 16646-1-AP), anti-F4/80 (total mouse macrophage marker; Abcam, ab16911), and anti-CD31 (JC/70A) (Thermo Fisher Scientific, MA5-13188). PAS staining utilized a PAS reagent (Sigma, 3952016) and a prepared 0.5% Periodic Acid Solution (Sigma, 395132), with positive protein expression indicated by brown staining. VM formation was identified using channels encircled by tumor cells exhibiting PAS+/CD31− staining. Scoring was performed by assessing the percentage of positive cells and staining intensity, and the resulting points were multiplied. Tumor specimens were categorized into three groups based on overall scoring: negative expression (0 to 1), weak expression (2 to 4) and high expression (6 to 12 points), resulting in total scores of 0 to 4 (low) or 6 to 12 (high). All evaluations were conducted independently by two pathologists who were blinded to patient identities and clinical outcomes.
