# Supplementary material for: ERα-dependent crosstalk between macrophages and cancer cells potentiates vasculogenic mimicry and M2 macrophage polarization in bladder cancer
Source: Cell Commun Signal. 2025 Jul 15;23:339. doi: 10.1186/s12964-025-02297-7 (PMC12261844; doi:10.1186/s12964-025-02297-7)
Supplement: Supplementary file 3 — Supplementary Material 3 [file 12964_2025_2297_MOESM3_ESM.docx]

Supplement Table 1

| ID | Forward primer | | Reverse primer |
| --- | --- | --- | --- |
| ESR1 | 5’- TTATGGGGTCTGGTCCTGTG-3’ | 5’- CATCTCTCTGACGCTTGTGC-3’ | |
| CDH5 | 5’-CCCACAGGCACGATCTGTT-3’ | 5’-GAGTCTCCAGGTTTTCGCCA-3’ | |
| GAPDH | 5’-AACGGATTTGGTCGTATTG-3’ | 5’-GGAAGATGGTGATGGGATT-3’ | |
| CDH5-ERE1 | 5’-ACACGTTTCCTGGGGAGCTA-3’ | 5’-ACACGTTTCCTGGGGAGCTA-3’ | |
| CDH5-ERE2 | 5’-ATGGACCACAGGAACAGTCG-3’ | 5’-GCACCATCTGCCAATTCCCT-3’ | |
| CDH5-ERE3 | 5’-ATCTCCTGAAGCCTCCCTGT-3’ | 5’-AGCGGGTTTAAGGTGCTTGT-3’ | |
| ARG1 | 5’-GTGGAA ACTTGCATGGACAAC-3’ | 5’-AATCCTGGCACATCGGGAATC-3’ | |
| CCL17 | 5’-GGAGCCATTCCCCTTAGAAAG-3’ | 5’-GCATTCTTCACTCTCTTGTTGTTG-3’ | |
| IL-1 | 5’-AAGATGTGCCTGTCCTGTGTC-3’ | 5’-GCTTGTCCTGCTTTCTGTTCTC-3’ | |
| IL-8 | 5’-CTGGACCCCAAGGAAAACTG-3’ | 5’-CCCTACAACAGACCCACACAAT-3’ | |
| CXCL-2 | 5’-AAACCGAAGTCATAGCCACACT-3’ | 5’-TCAGGAACAGCCACCAATAAG-3’ | |
| IL-10 | 5’-CTCCAAGAGAAAGGCATCTACAAA-3’ | 5’-GGGGTTGAGGTATCAGAGGTAATAA-3’ | |
| IL-17A | 5’-AATGAAACCCTCCCCAAAATAC-3’ | 5’-AATCTCCAAAGGAAGCCTGAGT-3’ | |
| IL-6 | 5’-GAGGATACCACTCCCAACAGACC-3’ | 5’-AAGTGCATCATCGTTGTTCATACA-3’ | |
| CXCL-10 | 5’-CCTCTCCCATCACTTCCCTACA-3’ | 5’-GGGTCAGAACATCCACTAAGAACA-3’ | |
| TGFB1 | 5’-AGTGCTACTTTGAGCGCTTCTC-3’ | 5’-GCCGAAGAGCTTCAGGAAGCAAGG-3’ | |
| CD163 | 5’-TTTGTCAACTTGAGTCCCTTCAC-3’ | 5’-TCCCGCTACACTTGTTTTCAC-3’ | |
| CD206 | 5’-GGGTTGCTATCACTCTCTATGC-3’ | 5’-TTTCTTGTCTGTTGCCGTAGTT -3’ | |
| CD68 | 5’-CTACTTTGCCATCCTTCA-3’ | 5’-GTGGTTTTGTGGCTCTTGGTA-3’ | |
| CD86 | 5’-CTGCTCATCTATACACGGTTACC-3’ | 5’-GGAAACGTCGTACAGTTCTGTG-3’ | |
| NOS2 | 5’-TTCAGTATCACA ACCTCAGCAAG-3’ | 5’-TGGACCTGCAAGTTA AAATCCC-3’ | |
| TNFα | 5’-CCTGTAGCCCACGTCGTAGC-3’ | 5’-AGCAATGACTCCAAAGTAGACC-3’ | |
| CCR7 | 5’-TGAGGTCACGGACGATTACAT-3’ | 5’-GTAGGCCCACGAAACAAATGAT-3’ | |
| PTEN | 5’-GTCAGAGGCGCTATGTGTATTA-3’ | 5’-TTAGCTGGCAGACCACAA-3’ | |
| hsa-miR-575 | 5’-GAGCCAGTTGGACAGGAG-3’ |  | |
| hsa-let-7b-3p | 5’-CTATACAACCTACTGCCTTCCC-3’ |  | |
| hsa-miR-449b-3p | 5’-CAGCCACAACTACCCTGCCACT-3’ |  | |
| hsa-miR-642a-5p | 5’-GTCCCTCTCCAAATGTGTCTTG-3’ |  | |
| hsa-miR-6501-5p | 5’-AGTTGCCAGGGCTGCCTTTGGT-3’ |  | |
| hsa-miR-423-5p | 5’-TGAGGGGCAGAGAGCGAGACTTT-3’ |  | |
| hsa-miR-4476 | 5’-CAGGAAGGATTTAGGGACAGGC-3’ |  | |
| hsa-miR-6740-3p | 5’-TGTCTTCTCTCCTCCCAAACAG-3’ |  | |
| hsa-miR-6754-3p | 5’-TCTTCACCTGCCTCTGCCTGCA-3’ |  | |
